# EgJUB1 and EgERF113 transcription factors as potential master regulators of defense response in *Elaeis guineensis* against the hemibiotrophic *Ganoderma boninense*

**DOI:** 10.1186/s12870-020-02812-7

**Published:** 2021-01-22

**Authors:** Nurshafika Mohd Sakeh, Siti Nor Akmar Abdullah, Mohammad Nazri Abdul Bahari, Azzreena Mohamad Azzeme, Noor Azmi Shaharuddin, Abu Seman Idris

**Affiliations:** 1 Institute of Plantation Studies, Universiti Putra Malaysia (UPM), 43400 Serdang, Selangor Malaysia; 2 Department of Agriculture Technology, Faculty of Agriculture, Universiti Putra Malaysia (UPM), 43400 Serdang, Selangor Malaysia; 3 Department of Biochemistry, Faculty of Biotechnology and Biomolecular Sciences, Universiti Putra Malaysia (UPM), 43400 Serdang, Selangor Malaysia; 4 Ganoderma and Diseases Research for Oil Palm Unit, Malaysian Palm Oil Board, No. 6, Persiaran Institusi, Bandar Baru Bangi, 43000 Kajang, Selangor Malaysia

**Keywords:** JUNGBRUNNEN 1, ERF113, SNBE motif, GCC-box, DRE/CRT, Hemibiotrophic

## Abstract

**Background:**

Hemibiotrophic pathogen such as the fungal pathogen *Ganoderma boninense* that is destructive to oil palm, manipulates host defense mechanism by strategically switching from biotrophic to necrotrophic phase. Our previous study revealed two distinguishable expression profiles of oil palm genes that formed the basis in deducing biotrophic phase at early interaction which switched to necrotrophic phase at a later stage of infection.

**Results:**

The present report is a continuing study from our previous published transcriptomic profiling of oil palm seedlings against *G. boninense*. We focused on identifying differentially expressed genes (DEGs) encoding transcription factors (TFs) from the same RNA-seq data; resulting in 106 upregulated and 108 downregulated TFs being identified. The DEGs are involved in four established defense-related pathways responsible for cell wall modification, reactive oxygen species (ROS)-mediated signaling, programmed cell death (PCD) and plant innate immunity. We discovered upregulation of *JUNGBRUNNEN 1* (*EgJUB1*) during the fungal biotrophic phase while *Ethylene Responsive Factor 113* (*EgERF113*) demonstrated prominent upregulation when the palm switches to defense against necrotrophic phase. EgJUB1 was shown to have a binding activity to a 19 bp palindromic SNBE1 element, WNNYBTNNNNNNNAMGNHW found in the promoter region of co-expressing EgHSFC-2b. Further in silico analysis of promoter regions revealed co-expression of EgJUB1 with TFs containing SNBE1 element with single nucleotide change at either the 5th or 18th position. Meanwhile, EgERF113 binds to both GCC and DRE/CRT elements promoting plasticity in upregulating the downstream defense-related genes. Both TFs were proven to be nuclear-localized based on subcellular localization experiment using onion epidermal cells.

**Conclusion:**

Our findings demonstrated unprecedented transcriptional reprogramming of specific TFs potentially to enable regulation of a specific set of genes during different infection phases of this hemibiotrophic fungal pathogen. The results propose the intricacy of oil palm defense response in orchestrating EgJUB1 during biotrophic and EgERF113 during the subsequent transition to the necrotrophic phase. Binding of EgJUB1 to SNBE motif instead of NACBS while EgERF113 to GCC-box and DRE/CRT motifs is unconventional and not normally associated with pathogen infection. Identification of these phase-specific oil palm TFs is important in designing strategies to tackle or attenuate the progress of infection.

**Supplementary Information:**

The online version contains supplementary material available at 10.1186/s12870-020-02812-7.

## Background

*Ganoderma boninense* is a pathogenic species which causes basal stem rot (BSR) disease in oil palm. Infected palms remain symptomless even though they are already physiologically impaired [1]. Half of the basal stem would have been destroyed by the fungus, compromising intake of water and nutrients, before the first symptom is observed [2, 3]. The fungus, *G. boninense* is recognized as a hemibiotroph, which established an intermediate lifestyle of biotroph before switching to necrotroph [4, 5]. The biotrophic phase involves colonization of host plant tissues and extraction of nutrients for survival by the pathogen while keeping the host cells intact [3]. Biotrophs thrive in the intracellular region between mesophyll cells through formation of haustoria that play important role in delivery of pathogenic effector proteins [6, 7]. The invasion requires minimal release of cell wall degrading enzymes (CWDEs) to allow loosening of the plant cell wall [8, 9].

Pathogen-associated molecular patterns (PAMPs) are elicitors such as bacterial flagellin and fungal chitin derived from the phytopathogens that are recognized by plant cell surface pattern recognition receptors (PRRs) leading to PAMP-triggered immunity (PTI) [10–12]. The susceptibility of plant host to microbial colonization relies on effectors secreted by the pathogens to deceive PAMPs recognition, thus suppressing PTI signaling [12, 13]. Breaching of the PTI remarks activation of a second-line of plant defense response termed as effector-triggered immunity (ETI). ETI comprises resistance (R) proteins to counteract a successful invasion of pathogens by recognizing virulence effectors (Avr) in host cells [14]. ETI, accompanied by increased signaling of phytohormones including lipid-based jasmonic acid (JA) and gaseous ethylene (ET) stimulate the production of ROS [15]. The ROS induces a hypersensitive response (HR) at sites of infection to limit the invasion of pathogens [16].

Unfortunately, the accumulation of ROS and induced programmed cell death (PCD) created a favorable environment for necrotroph to intensify infection strategy [17]. It was postulated that the increasing pressure by plant defense responses results in switching of the pathogen infection mode from biotrophy to necrotrophy but the time taken differs between specific host-pathogen interaction [18]. Hemibiotrophs may require extended periods of biotrophic phase to establish colonization and once sufficed, transition to necrotrophic phase is rapid [19]. Necrotrophs secrete phytotoxic compounds and excessive CDWEs to induce host cell death [20]. The dynamic intermediate lifestyle of hemibiotroph enables manipulation of the host plant defense mechanisms which ultimately result in the plant succumbing to the infection. Salicylic acid (SA) signaling which often functions antagonistically to JA-ET signaling has been widely studied to determine the fine-tuning against biotrophic or necrotrophic infection [21–23]. However, there is poor molecular information available explaining the defense regulation by transcription factors (TFs) during transitions of biotrophic to necrotrophic state. Our previous transcriptomic profiling via high-throughput RNA-seq analysis has pointed out the counter-act defense mechanisms executed by plants during the transition of biotrophic to necrotrophic phase [5]. In the attempt of identifying key defense pathways that are transcriptional regulated, the next generation sequencing (NGS) data set was mined for TFs responsible for triggering the downstream responses.

TFs are the ‘master switches’, which regulate the expression of a large set of genes initiated by unique signaling networks in response to stresses [24]. Modulation of defense response gene expression may vary depending on the intensity and intricacy of multiple stresses [25]. There are six major TF families involved in plant defense response including MYB, bHLH, AP2/ERF, NAC, bZIP and WRKY [26]. Regulation by TFs is crucial to mediate the transcriptional reprogramming which includes induced expression of defense-related genes responsible in the production of antifungal proteins or the antimicrobial secondary metabolites known as phytoalexins [27]. Intriguingly, plants also display cross-tolerance phenomena in which a single type of stress may trigger a multitude of tolerant levels to different stresses [28]. For instance, heat stress transcription factors (HSFs) play an important role as a master regulator of defense response under multiple stresses [29, 30]. Over-expressed *HSFs* confer resistance against dehydration, bacterial and oomycetes infections and improve yield under water-limited conditions [31].

TFs bind to *cis*-acting elements located in promoters of either other mediator TFs or downstream target genes which results in up- or down-regulation of their expression [32–34]. NAC TFs are recognized as master regulators for secondary cell wall biosynthesis mediated by MYB TF through the formation of NAC-MYB-CESA signaling cascade [35]. In addition, bHLH-MYB association was involved in the biosynthesis of flavonoids secondary metabolites under phytohormones signaling, wounding and fungal interaction [36, 37]. Interaction to specific DNA sequences (binding motifs) is dependent on the DNA binding domain (DBD) of TFs [38]. Binding preferences of TF during biotic or abiotic stress such as ERFs have been suggested to correlate with the composition of amino acid sequences in DBD [38, 39]. The presence of multiple *cis*-acting elements in the promoter region contributes to overlapping roles in development and/or defense against multiple stresses of the expressed proteins [26].

In addition to biotic or abiotic stresses [40], in this study, we reported that the regulation of TFs is dependent on modes of pathogen infection (biotrophic and necrotrophic). Only *EgUNE10* TF and a few TF families have been reported to regulate defense against later stages of *G. boninense* infection [4, 41, 42]. However, there is no comprehensive report on transcriptomic profiling of defense-related TFs during different infection phases of hemibiotroph in plants. Thus, this study is the first attempt to recognize specific TFs as ‘key’ biomarkers involved in transcriptional switching from biotrophic to necrotrophic infection phase based on early oil palm-*G. boninense* interaction. Their potential targeted defense response pathways that distinguished the two phases are discussed based on the identification of specific motifs interacting with the newly discovered TFs. The findings might allow a more effective disease management strategy to attenuate the progress of *G. boninense* infection of oil palm and prevent the spread of the disease.

## Results

### *Elaeis guineensis* defense-related transcription factors and biomarkers of biotrophy-necrotrophy switch

**Fig. 1:**
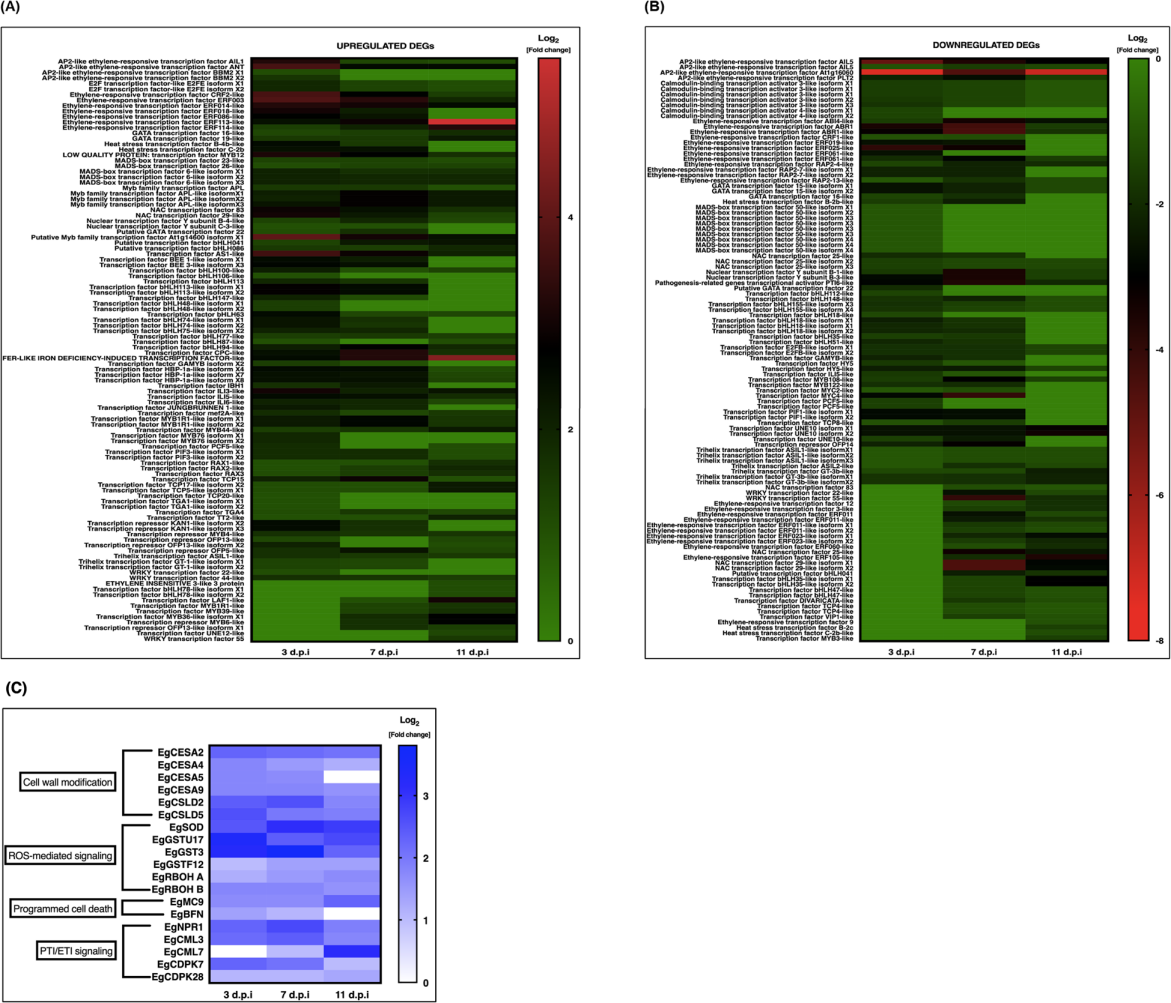
Time course of differentially expressed genes (DEGs) during early interaction with *Ganoderma boninense.* **a** Heat map clustering of upregulated transcription factors. Colored blocks indicate ascending expression level from green (0) to red (6). **b** Heat map clustering of downregulated transcription factors. Colored blocks indicate descending expression level from green (0) to red (− 8). **c** Heat map clustering of upregulated DEGs commonly expressed during biotic interaction. Colored blocks indicate ascending expression level from white (0) to blue (4). For RNA-seq data analysis, two biological replicates which each consisted of pooled RNA provided equally from six constituent seedlings were used. Each heatmap data was constructed using an average of pooled biological replicates. Pairwise comparison of RNA-seq data between control (untreated) and *Ganoderma*-treated was evaluated according to cut-off values of log_2_ fold change (FC) ≥ |1.0| and *P*-value < 0.01

In order to identify the TF families involved in the early defense of oil palm against *G. boninense*, transcriptomics analysis of *Ganoderma*-treated root tissues at 3, 7 and 11 d.p.i was carried out. We identified 106 of upregulated and 108 of downregulated TFs upon early interaction with *G. boninense* (Fig. 1a and b). The pairwise comparison was constructed between control against time course treatments using stringent cut-off values of log_2_ fold change (FC) ≥ |1.0| and *P*-value < 0.01. We have previously reported the transition of biotrophic to necrotrophic defense mechanism, based on qPCR preliminary screening using defense-related molecular biomarkers *EgPR1* (biotrophic) and *EgMYC2* (necrotrophic) [5]. Based on the report, the expression profile of defense-related TFs was identified as biotrophic-regulated at 3 days post-infection (d.p.i) while necrotrophic-regulated at 11 d.p.i, with intermediate at 7 d.p.i. We found that the expression patterns of the genes showed either decreasing or increasing over time. The highest TF families upregulated during early interaction with *G. boninense* were mainly *bHLH* > *MYB* > *AP2/ERF*, followed by *bZIP* > *MADS* > *TCP* > *OFP* > *NAC* > *GATA* > *HSF* > *NFY* > *E2F* > *WRKY> EIN/EIL*. On the other hand, the highest downregulated families of TF were found to be mainly *AP2/ERF* > *bHLH* > *MYB* followed by *MADS* > *CAMTA* > *NAC* > *TCP* > *GATA* > *bZIP* > *HSF* > *NFY* > *E2F* > *WRKY* > *OFP*. The families of oil palm’s TFs involved during defense response against *G. boninense* were found to be the same but involving different members in both upregulated and downregulated DEGs. Distinctively, *EIN/EIL* and *CAMTA* TF family were only found in upregulated and downregulated DEGs, respectively. *AtCAMTA3* and *AtCAMTA4* were reported to negatively regulate plant defense response under SA-mediated signaling pathway against obligate biotroph [43, 44].

To further understand plant response against *G. boninense* interaction, four common pathways regulating defense mechanisms were identified from the RNA-seq data (Fig. 1c). Even though not all genes have been discovered in the pathways, important genes associated with the different pathways have been identified among the DEGs as presented. Reported genes involved in cell wall modification, ROS-mediated signaling, PCD and plant innate immunity were all differentially expressed, indicating active regulation of defense response in *E. guineensis* against hemibiotroph *G. boninense*. The expression patterns of selected biotic stress-related genes were important to distinguish the defense mechanisms executed by the plants during two different phases of biotrophic and necrotrophic infection.

Six genes of *Cellulose synthase* (*CESA*) and *Cellulose synthase-like D* (*CSL*) were reported in cell wall modification including *EgCESA2*, *EgCESA4*, *EgCESA5*, *EgCESA9*, *EgCSLD2* and *EgCSLD5* demonstrated similar expression patterns with high upregulation at 3 d.p.i that successively decreased across time. Three out of six genes involved in ROS-mediated signaling including *Superoxide dismutase* (*EgSOD*), *Glutathione S-transferase* (*EgGSTF12*) and *Respiratory burst oxidase homolog* (*EgRBOHA*) were increasingly expressed from 3 d.p.i to 11 d.p.i, whilst *EgGSTU17*, *EgGST3* and *EgRBOHB* demonstrated decreasing expression patterns. Two genes reported in PCD, *Metacaspase 9* (*EgMC9*) and *Bifunctional nuclease* (*EgBFN*) showed antagonistic expression patterns of increasing and decreasing regulation, respectively. Five genes involved in plant innate immunity of PTI/ETI signaling were found to be differentially expressed. Three of the genes including *NONEXPRESSOR OF PATHOGENESIS-RELATED GENE 1* (*EgNPR1*), *Calmodulin-like 3* (*EgCML3*) and *Calcium-dependent protein kinase 7* (*EgCDPK7*) were upregulated the highest at 3 d.p.i and 7 d.p.i before subsequently declined. The other genes which were *EgCML7* and *EgCDPK28* were successively upregulated with the highest expression at 11 d.p.i. The reliability of RNA-seq data has been validated in our previous report [5] using representative upregulated and downregulated DEGs with different levels of fold change compared to untreated seedlings (control).

### Expression patterns of transcription factors associated with defense response against *Ganoderma boninense*

**Fig. 2:**
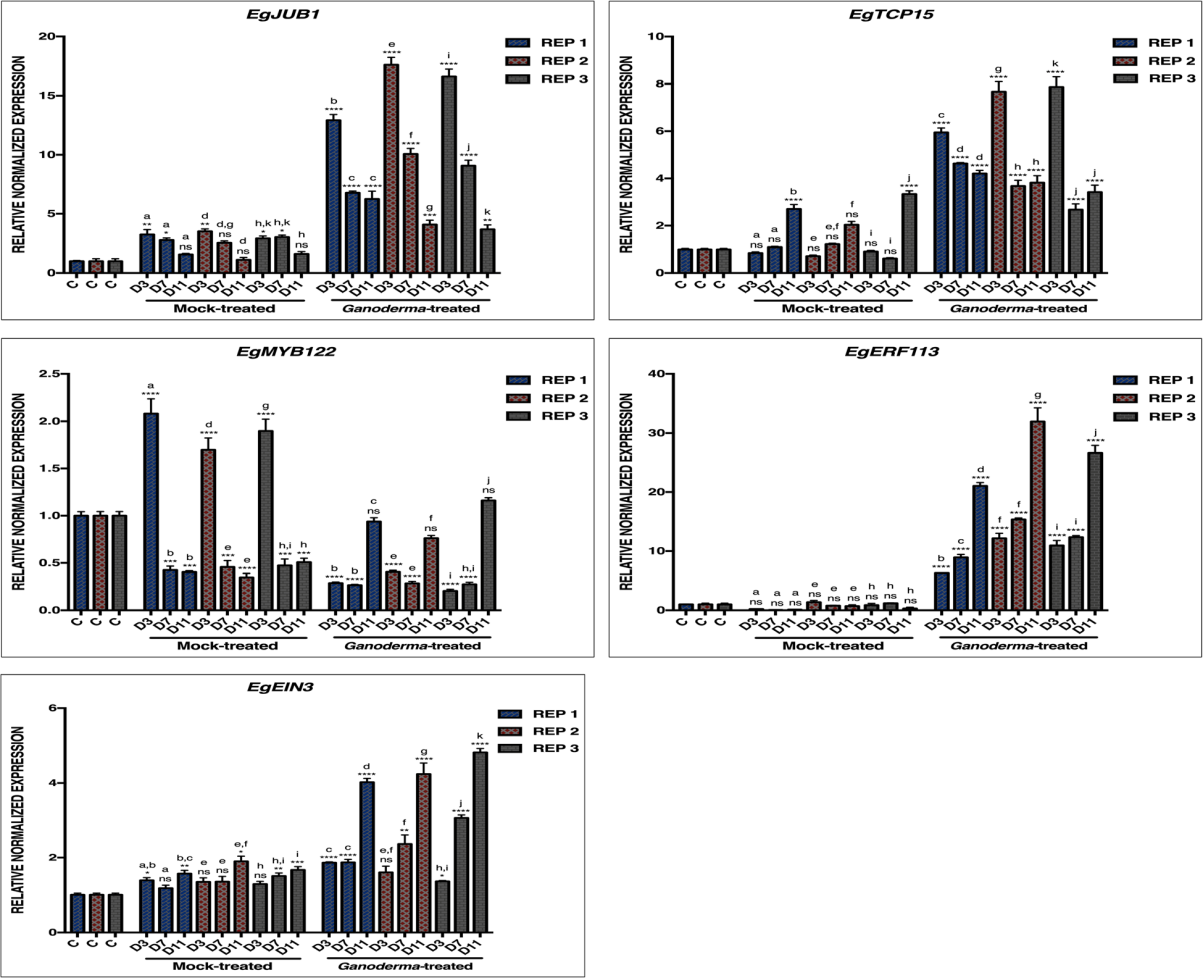
Expression patterns of transcription factors in response to *Ganoderma boninense* infection at different time points. The expression patterns of each gene were normalized by the three most stable reference genes; *EgGAPDH2*, *EgNADH5* and *Egß-actin* expression levels. Real-time PCR was carried out on control (C), mock-treated (MT) and *Ganoderma-*treated (GT) oil palm root samples at 3, 7 and 11 days post-inoculation (d.p.i). The data included three biological replicates of root samples of oil palm seedlings. Error bars represent the mean ± SEM of three technical replicates of each sample. The statistical analyses were performed by comparing expression levels of different treatments at all time points to control using one-way ANOVA analysis followed by Tukey’s test for comparison between treatments. Significantly different expression level as compared to control are measured according to **P < 0.01, ***P < 0.001 and ****P < 0.0001. ns is defined as not significant

The RNA-seq data was generated by pairwise comparison between control (untreated) and *Ganoderma-*treated (GT) samples across time course treatments (3,7 and 11 d.p.i). In order to screen for defense-related TFs specific to biotic stress from a large set of candidate genes, multiplex semi-quantitative PCR was performed by comparing relative amounts of mRNA between groups (GT and mock-treated (MT)) based on the band intensity of amplicons (data not shown). qPCR analysis was able to further distinguish five TF candidates regulated under biotic and/or abiotic stresses by comparing the expression patterns of GT and MT samples, respectively at designated time points against control (Fig. 2). Biotrophic-associated TFs, *EgJUB1* and *EgTCP15* were highly upregulated at 3 d.p.i before declining in GT samples. However, *EgTCP15* also exhibited significant upregulation in MT samples at 11 d.p.i. TCP15 was reported in both developmental and stress response pathways under SA-mediated signaling, enhanced by interaction with NPR1 [45, 46]. The expression levels in MT samples at all time points were significant for all analyzed genes except for *EgJUB1*, which indicates the *EgJUB1* specific role during the biotrophic infection phase under biotic stress (*Ganoderma* infection).

Meanwhile, the qPCR analysis on candidates of necrotrophic-associated TFs revealed that *EgERF113*, *EgEIN3* and *EgMYC2* were highly upregulated at 11 d.p.i on GT samples. Both *EIN3* and *MYC2* are JA-dependent which were upregulated and downregulated in the RNA-seq data, respectively. The expression of these genes is known to be mutually exclusive whereby MYC2 relies on co-actions of JA-abscisic acid (ABA) while *EIN3* regulates plant defense response through JA-ET signaling [47, 48]. *EgERF113* which demonstrated non-significant expression on MT samples (abiotic stress) at all time points were selected for further characterization as a novel potential candidate of necrotrophic-specific TF.

### EgJUB1 binds to novel SNBE motif during biotrophic infection

**Fig. 3:**
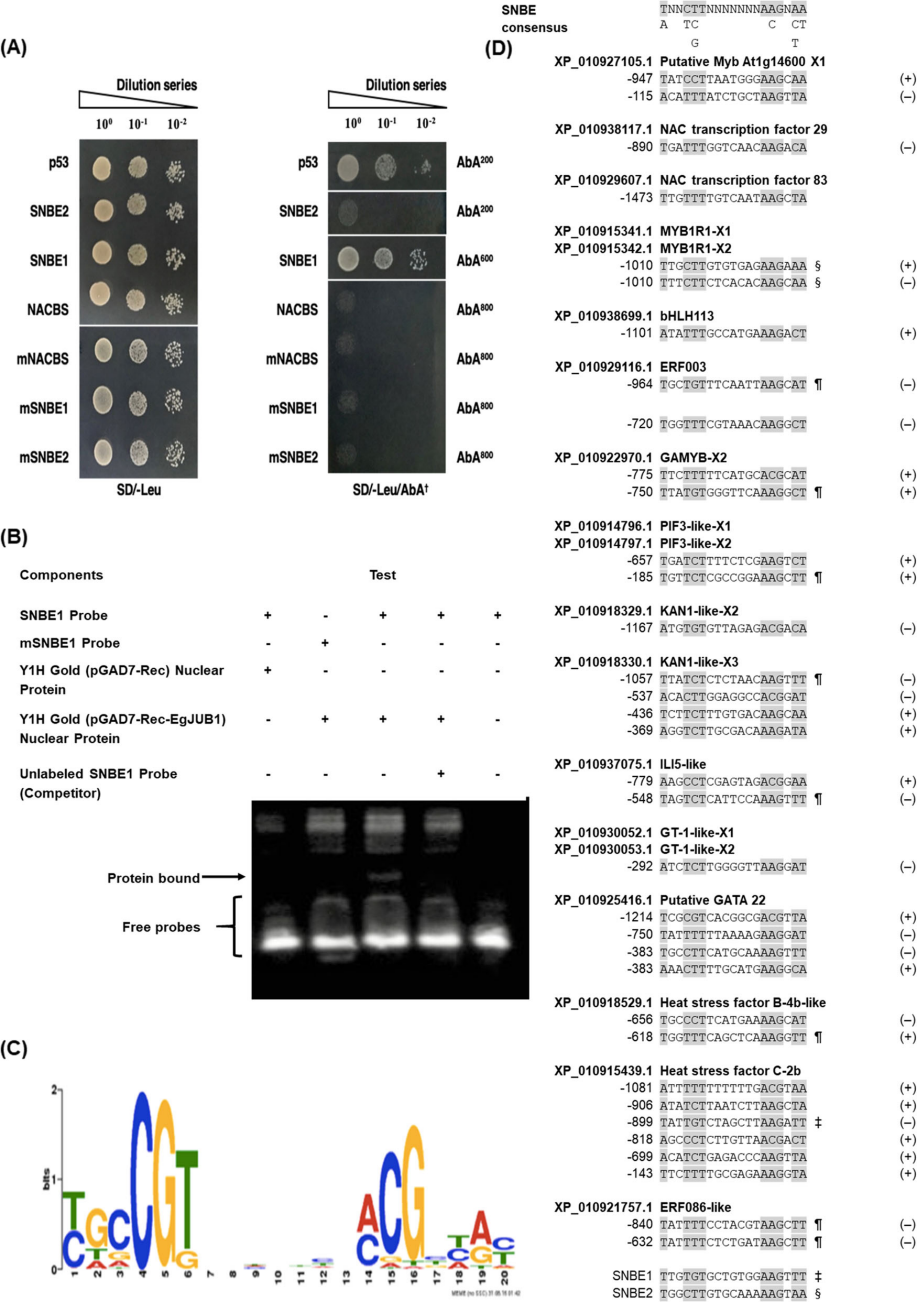
EgJUB1 interacts with secondary wall NAC binding element (SNBE) during defense response against biotrophic infection. **a** Yeast One-Hybrid analysis reveals interaction of EgJUB1 with tandem repeats of SNBE1 motif. Negative interaction was observed with both NAC binding site (NACBS) and SNBE2 motifs. Transformed yeast cells were cultured on SD/−Leu/AbA^†^, wherein ^†^ denotes optimized concentration of antibiotic Aureobasidin A (AbA) for each motif. **b** EMSA shows direct binding of EgJUB1 to SNBE1 motif. **c** Prediction of binding motifs for EgJUB1 derived from Plant Transcription Factor Database (PlantTFDB) version 5.0. http://planttfdb.cbi.pku.edu.cn/. **d** Putative SNBE sequences identified from 1.5-kb promoters of EgJUB1 direct targets. The number shown on the left of each sequence is the position of the first nucleotide relative to the start codon. The plus or minus symbol on the right of each sequence indicates first nucleotide of SNBE sequences from the forward or reverse strand of the target’s DNA. ‡ indicates SNBE1 core motif and § indicates SNBE2 core motif tested in the study. ¶ represents SNBE1 core motif used in the present study, with single nucleotide change. X is defined as an isoform

Characterization of EgJUB1 TF against *G. boninense* infection was carried out via Y1H assay and EMSA. Three potential binding motifs; one NAC binding site (NACBS) and two secondary wall NAC binding elements (SNBEs) with respective mutants were tested on EgJUB1 (Fig. 3). NACBS is the established binding motif for JUB1 TF during abiotic stress while SNBEs are the novel target motifs tested in the present study. The SNBE consensus sequence was identified as WNNYBTNNNNNNNAMGNHW, whilst NACBS was RRYGCCGT. Y1H assay demonstrated positive interaction with SNBE1 motif but not to NACBS and SNBE2. The results indicate regulation of the alternative pathway by JUB1 in enhancing resistance during biotic stress. The positive binding with one of the SNBE1 motif indicates that the binding affinity is dependent on the core motifs of the SNBE consensus sequence, whereby four nucleotides change on the core motif of SNBE1 resulted in no interaction with SNBE2 (Fig. 3a). Colony PCR was carried out on five positive clones and sequencing was performed using T7 promoter primer of pGADT7-Rec vector to confirm genuine positive interaction. p53-AbAi yeast reporter vector was used as a positive control in the Y1H assay. We further confirmed the protein-DNA interaction via EMSA by testing the binding of nuclear protein extracted from a positive clone of Y1H assay on biotinylated DNA probes. A shifted band was observed upon testing with a biotinylated DNA probe. Unlabeled probe (molar excess 200-fold) was able to compete effectively for binding with biotinylated target DNA probe. The inability of the mutated fragment to compete with the biotinylated target DNA probe revealed specific binding of EgJUB1 to SNBE1 (Fig. 3b). SNBE consensus sequence is the predicted DNA binding motif of JUB1 (Fig. 3c), based on Plant Transcription Factor Database (PlantTFDB), but thus far there is no reported evidence to support this.

Here, we listed SNBE motifs explored in the 1.5 kb promoter regions of TFs co-expressed with *EgJUB1* during the biotrophic phase (Fig. 3d). From the RNA-seq data, EgJUB1 showed significant upregulation at 3 and 7 d.p.i but not at 11 d.p.i, based on statistical analysis of cut-off values log_2_ fold change |1.0| and *P*-value < 0.01. Hence, TFs with a similar pattern of expression profile using the same statistical analysis are categorized as co-expressing with EgJUB1. The core motif of the SNBE1 sequence was identified in the promoter region of *EgHSFC-2b*, suggesting direct regulation of EgJUB1 with the *EgHSFC-2b*. We highlighted the promoter regions of a few TFs co-expressing with *EgJUB1* with a single nucleotide change in the SNBE1 core motif tested in the present study at the 5th or 18th position, including *EgHSFB-4b*, *EgGAMYB-X2*, *EgERF003*, *EgKAN1-like-X3*, *EgILI-5-like*, *EgERF086-like*, *EgPIF3-X1* and -*X2*. It is plausible that changes of single nucleotide on SNBE1 core motif, still maintain the ability of EgJUB1 to activate these TFs. Changes of two and more nucleotides in the core motif of SNBE1 are expected to result in a significant decline in binding affinity [49], which merit analysis in more detail.

### EgERF113 binds to both GCC and DRE/CRT motifs during necrotrophic infection

**Fig. 4:**
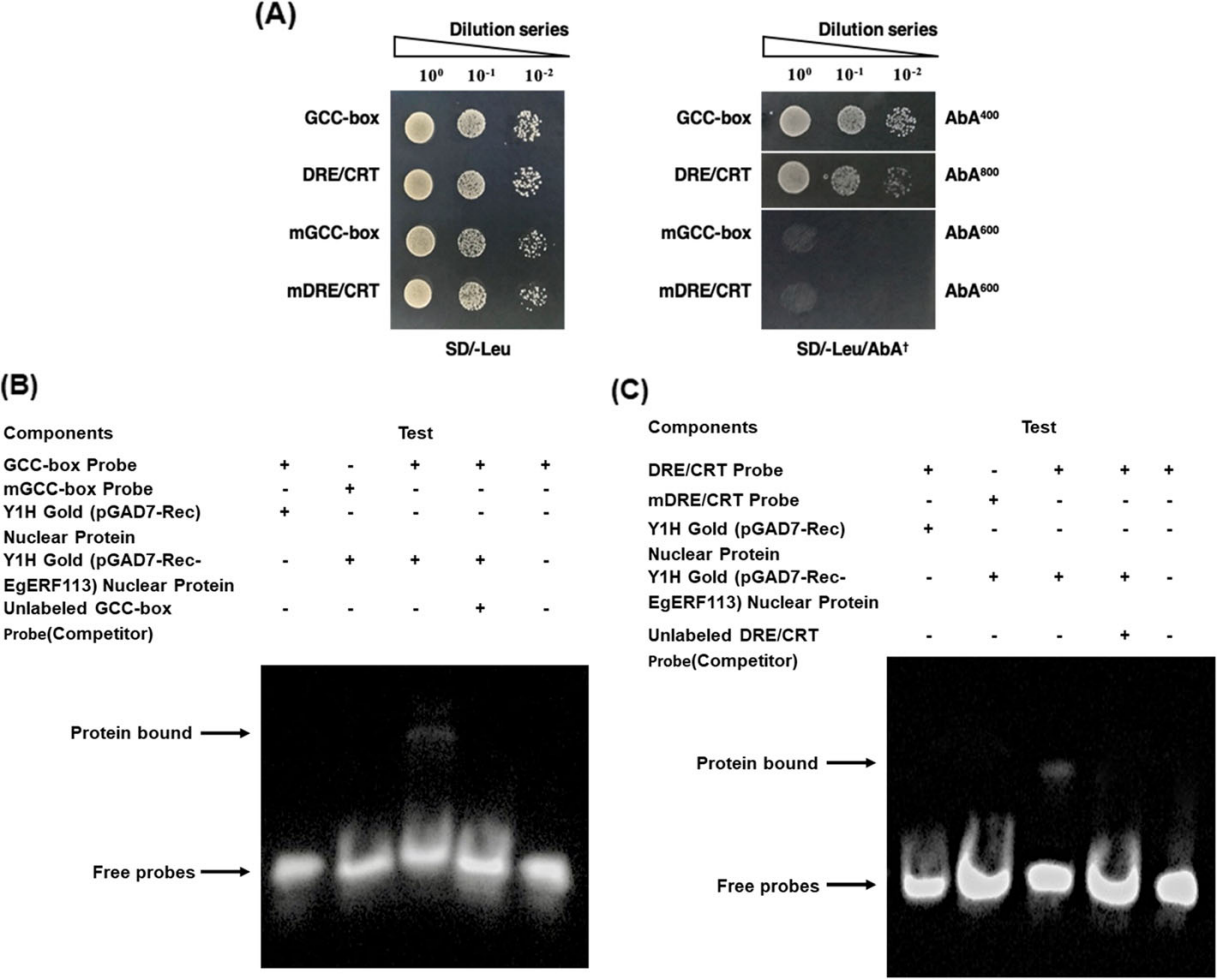
EgERF113 interacts with both GCC-box and DRE/CRT elements during defense response against necrotrophic infection. **a** Yeast One-Hybrid analysis reveals positive interactions with both GCC-box and DRE/CRT motifs. Transformed yeast cells were cultured on SD/−Leu/AbA^†^, wherein ^†^ denotes optimized concentration of antibiotic Aureobasidin A (AbA) for each motif. EMSA shows specific binding of EgERF113 to (**b**) GCC-box and (**c**) DRE/CRT motifs

EgERF113 TF was tested via yeast one-hybrid (Y1H) assay and electrophoretic mobility shift assay (EMSA) (Fig. 4) on two AP2/ERF DNA binding preferences which were GCC-box also known as Ethylene-Response Element (ERE) and Dehydration Response Element/C-Repeat (DRE/CRT). The GCC-box and DRE/CRT motifs in the study share a 6-bp core sequence of GCCGMC. Our findings revealed recognition of EgERF113 by both motifs in the Y1H assay. The binding affinity of EgERF113 with both binding motifs was supported by the EMSA results. Unlabelled probes of GCC-box and DRE/CRT (molar excess 200-fold) were able to compete with respective biotinylated target DNA probes. No shifted band was observed on mutated fragments which proved binding specificity of EgERF113 with both GCC-box and DRE/CRT motifs. The findings suggest that EgERF113 can regulate stress-related genes harboring GCC-box and/or DRE/CRT in their promoter region.

### EgJUB1 and EgERF113 are localized in the nucleus

**Fig. 5:**
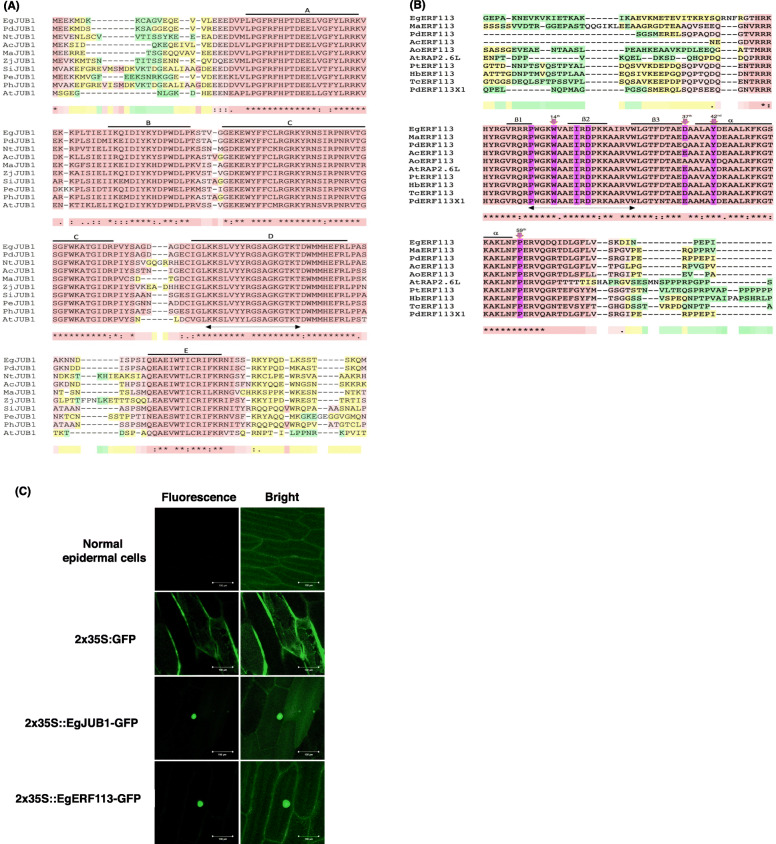
Sequence characterization and nuclear localization of EgJUB1 and EgERF113. **a** Multiple sequence alignment of the deduced amino acid sequence of EgJUB1 protein with other JUB1 in different species. Highly conserved NAC subdomains (A to E) are indicated by black lines. **b** Multiple sequence alignment of the deduced amino acid sequence of EgERF113 protein with other ERF113 in different species. Pink shadings indicate specific amino acids for binding to GCC-box and DRE/CRT motifs. Structure-based alignment was constructed using mode Expresso of T-Coffee software http://tcoffee.crg.cat/apps/tcoffee/do:expresso. The asterisks indicate fully conserved residues. The colons indicate the conservation of strong group. The full stops indicate the conservation of weak group. The dashes indicate no consensus. The red shadings indicate reliable and consistent alignment. Yellow and green shadings indicate average reliability while blue shadings indicate very poor alignment. The putative nuclear localization signal is shown by double-headed arrow below the sequences. The deduced amino acids sequences of JUB1 and ERF113 are derived from different species; *Ananas comosus* (Ac), *Arabidopsis thaliana* (At), *Asparagus officinalis* (Ao), *Elaeis guineensis* (Eg), *Hevea brasiliensis* (Hb), *Musa acuminate* (Ma), *Nicotiana tabacum* (Nt), *Panicum hallii* (Ph), *Phalaenopsis equestris* (Pe), *Phoenix dactylifera* (Pd), *Populus trichocarpa* (Pt), *Setaria italic* (Si), *Theobroma cacao* (Tc) and *Ziziphus jujube* (Zj). **c** Subcellular localization of EgJUB1 and EgERF113 in onion epidermal cells. EgJUB1-GFP and EgERF113-GFP were expressed in the nuclei of cells and fluorescence signals were visualized with confocal microscope. Scale bars = 100 μm

Analysis of the deduced amino acid sequence revealed the NAC binding domain of EgJUB1 with five highly conserved subdomains A to E at the N-terminal region. The C-terminal region showed a highly conserved domain between different species which suggested its possible role as a transcriptional activator, repressor, or in binding to other proteins. Putative nuclear localization signals (NLS), KKSLVYYLGSAGKGTKT was identified in subdomain D (Fig. 5a).

The DBD of EgERF113 TF consists of three-stranded ß-sheets and one α-helix running almost in parallel. Analysis of the deduced amino acid sequence of EgERF113 proved the presence of tryptophan (W) amino acid at 14th position of the DBD which explained the binding specificities to both GCC-box and DRE/CRT motifs. An additional of one amino acid at 24th position of DBD results in 61 amino acids long and binding specificity to GCC-box only [39]. However, AP2/ERF DBD of EgERF113 lacks this additional amino acid. Putative NLS PWGKWAAEIRDPRKAIRV was identified in ß-sheets (Fig. 5b).

To determine the subcellular localization of EgJUB1 and EgERF113 proteins, we utilized the *Agrobacterium*-mediated transformation for transient expression of green fluorescent protein (GFP) in onion epidermal cells. As shown in Fig. 5c, both GFP-labelled EgJUB1 and EgERF113 could co-accumulate in the nucleus. The fusion protein of mGFP was fused with the C-terminal of the TFs. The observation is consistent with the putative role of these proteins acting as TFs.

## Discussion

Upon assaulted by pathogens, plants respond by activation of intricate defense systems. Depending on the nature of the pathogens, biotrophic and necrotrophic infections are fundamentally different in terms of their infection approach, effector proteins, and the host defense response [50]. Thus, tackling the disease based on the infection stage, hypothetically should be able to save or at least prolong the life span of *Ganoderma*-infected palms. Identifying the infection at the biotrophic phase may help planters to take suitable disease management strategies to prevent the disease from transition to the more chronic necrotrophic phase. Meanwhile, infected palms at the necrotrophic phase may be treated with more intensified practice such as using chemical fungicide. Differentiation of biotrophic and necrotrophic TFs were based on established defense-related biomarkers in known defense mechanisms such as plant innate immunity and HR that can distinguish these two phases. In this study, we demonstrated that *EgJUB1* potentially plays a key role as a biotrophic-specific, and *EgERF113* as a necrotrophic-specific transcriptional regulator, during early oil palm-*G. boninense* interaction.

### EgJUB1 likely mediates defense response during biotrophic phase through SNBE motif

The expression profile of *EgJUB1* observed in RNA-seq was validated via qPCR which suggested its role in host defense regulation during the biotrophic phase of *G. boninense* infection independent of abiotic stress. JUB1 was first linked to homeostasis of oxidative stress particularly related to hydrogen peroxide (H_2_O_2_) signaling. It binds to *cis-*element that serves as the NACBS in the promoter region of *DREB2A* TF for tolerance to abiotic stresses [51]. DREB2A TF binds directly to the DRE sequence of drought-stress responsive genes, including HSFs but the mechanisms are still unclear [52]. AtJUB1 (ANAC042) was also reported as a key TF that induces camalexin expression, a major phytoalexin of *Arabidopsis* against bacterial pathogen [53]. A more recent study revealed induced expression of *NAC042_5*, an orthologue of *AtJUB1* in response to biotrophic fungus *Erysiphe necator* [54]. The *JUB1* which acts independently of SA, was induced specifically during pathogen colonization. Intriguingly, oil palm *EgJUB1* was found to co-express with two candidate *EgTGA1* and *EgNPR1* orthologs. Our results were more in line with the SA-dependent master regulator of *NPR1*, a cofactor of *TGA1* reported by [55], which induces *Pathogenesis related* (*PR*) genes [56] during the biotrophic phase.

We are reporting for the first time induced expression of *EgJUB1* under pathogen challenge, regulating defense-related gene(s) harbouring SNBE binding motif. SNBE motif is composed of an imperfect palindromic 19-bp sequence which can be present in various targets’ promoters including TFs and downstream genes involved in secondary cell wall biosynthesis, cell wall modification as well as PCD [57]. Binding to the 19 bp SNBE (A/T)NN(C/T)(C/G/T)TNNNNNNNA(A/C)GN(A/C/T)(A/T) consensus sequence is critical at the 9 core nucleotides, regardless of mutation on the other nucleotides [49]. They reported that mutation(s) on these 9 core nucleotides causes reduced and/or elimination of the transcriptional activation, on the contrary changes in the other non-critical nucleotides enhance the binding affinity.

It was discovered from our study that EgJUB1 directly regulates *EgHSFC-2b* to promote resistance against the biotrophic phase through the SNBE1 motif. The SNBE1 motif tested in this study consists of the nucleotides G and T at the 5th and 18th position of the core motif, respectively. Based on the report by Zhong et al. [49], the binding affinity can still be maintained if a single nucleotide in the core motif of the SNBE consensus sequence is changed, however, changes of two and more nucleotides may reduce the binding affinity significantly. Thus, it is most likely that EgJUB1 is still able to bind to the promoters of the listed oil palm TFs (Fig. 3d), *EgHSFB-4b*, *EgGAMYB-X2*, *EgERF003*, *EgKAN1-like-X3*, *EgILI-5-like*, *EgERF086*-*like*, *EgPIF3-X1* and -*X2*, which harbour single nucleotide change at either the 5th or the 18th position of the SNBE1 core motif. Among these TFs, heat shock factors *EgHSFC-2b* and *EgHSFB-4b* were identified. Interestingly it is well known that HSFB is involved in transcriptional reprogramming during stress response [29]. Thus, defense mechanisms of oil palm against *G. boninense* may be channelled through the HSF pathways.

Our findings are in line with a recent study which observed high up-regulation of *HSF* and *heat shock proteins* (*HSPs*) against biotrophic fungus [58, 59]. HSF was also reported during bacterial infection which directly regulated *Enhanced Disease Susceptibility 1* (*EDS1*) and *PR4* under SA-mediated signaling [60].

### Proposed defense-related pathways regulated by *EgJUB1* co-expressing genes

Here, we report that high expression of *EgCESA4*, *EgCESA9* and *EgCSLD2* correlates with the expression of *EgGAMYB-X2* TF during the biotrophic phase (3 and 7 d.p.i) before a subsequent decline in expression. GAMYB TF interacts with GAMYB binding motif to activate downstream genes [61]. The GAMYB motif was found in the promoter region of *CESA* responsible for secondary cell wall cellulose biosynthesis [35, 62]. Consistently**,** *CESA4*, *CESA7* and *CESA9* were reported as regulators of secondary cell wall cellulose synthesis [63]. Besides, local cell wall reinforcement by *CSLD2* has been proven under the biotroph challenge of powdery mildew fungus (Douchkov et al., 2016). Thus, it is strongly postulated that EgJUB1 binding to SNBE1 motifs in the promoter regions of *EgGAMYB-X2* activates oil palm defense response through regulation of secondary cell wall biosynthesis.

Increased production of ROS accompanied with PCD [7] provides evidence on the occurrence of HR. In the current study, genes regulating antioxidant enzymes *EgSOD*, *EgGSTU17*, *EgGST3* and *EgGSTF12* were highly upregulated during early interaction with *G. boninense*. We also observed the expression of *EgNPR1* and *EgTGA1* which have been recognized to be overexpressed exclusively during biotrophic attack under SA-mediated signaling pathway [64, 65]. The co-actions of *EgNPR1* and *EgTGA1* result in upregulation of *EgPR1* which has been proven as a biotrophic marker in our previous report [5].

### EgERF113 likely mediates defense response during necrotrophic phase through GCC-box and DRE/CRT motifs

MYC2 relies exclusively on co-actions of JA-ABA branch response to regulate resistance against insects and wounding by repressing the JA-ET branch [48]. Although *EgMYC2* was preliminarily screened as a necrotrophic biomarker [5], the TF is categorized as a downregulated DEG in our RNA-seq data. This can be explained by elevated expression of ethylene insensitive *3* (*EgEIN3*) which then may activate *EgERF113* through ET regulation. Binding of ET to its receptors inactivates CONSTITUTIVE TRIPLE RESPONSE 1 (CTR1) which in turn release repression on EIN2 activity and stabilizes EIN3 and EIN3-LIKE1 (EIL1) within the nucleus [66]. As a result, *ERFs* are activated and the ERF TFs modulate transcriptional activity of developmental as well as stress-induced responsive genes [67]. Master regulators of ethylene signaling pathways, *EIN3* and *EIN3-like* (*EIL*) have been proven to modulate a multitude of cascades of downstream transcriptional responses [47, 68]. De-repression of *EIN3/EIL* from JA-ZIM domain (JAZ) activates JA-ET signaling that positively regulates transcriptional activations of development as well as defense response against necrotrophic pathogens [47, 69]. The findings demonstrated that oil palm establishes resistance against early necrotrophic through co-actions of JA-ET, rather than JA-ABA.

Here, we propose the regulation of defense response against necrotrophic phase of *G. boninense* through multi-cascades activation of JA-ET branch leading to overexpression of *EgERF113*. *ERF113*, also recognized as *RELATED TO APETALA2.6-LIKE* (*RAP2.6 L*) which is closely related to *ERF108* (*RAP2.6*) was found to be responsive to abiotic stresses (salinity, heat and drought) as well as stress hormones, particularly JA and/or ET [70]. ERF108 has been reported in JA-induced defense response against wounding and pathogens [71, 72]. Similarly, ERF113 was shown to be induced upon wounding [73, 74], as well as pathogens infection [75, 76].

We are the first reporting on EgERF113 with binding preferences for GCC-box and DRE/CRT motifs. AP2/ERFs are known to have multiple conserved DNA binding preferences [77]. However, DREBs are typically known to recognize DRE/CRT conferring resistance against abiotic stresses whilst ERFs bind to GCC-box promoting defense against biotic stresses. ERF113 was reported to bind to the GCC-box [76], but not tested on DRE/CRT motif. To date, only a few ERFs were reported with binding preferences on both GCC-box and DRE/CRT conferring resistance against pathogens attack [78–81]. Binding to GCC-box and DRE/CRT motifs that are present in plant defensin (e.g *PDF1.2*) as well as *PR* genes, activates defense-related genes [82, 83]. Kaur et al. [83], also reported DRE/CRT elements in the promoter region of calcium-responsive genes. Calcium ion (Ca^2+^) signaling plays paramount importance in the defense mechanisms of plants in perceiving invading pathogens [84].

Phukan et al. [39] have studied the divergent of AP2/ERF TF DNA-binding specificities based on sequence characterization. Their findings suggested glutamic acid (E) at the 20th and alanine (A) at the 48th positions as identified in EgERF113 in the 60 amino acids long DBD denote binding specificity to DRE/CRT motif. EgERF113 also showed conservation of amino acids for GCC-box binding at the 10th, 18th, 20th, 37th and 59th positions. Although the specific amino acid in DBD that regulates binding of TFs to both GCC-box and DRE/CRT motifs has not yet been confirmed, replacing phenylalanine (F) at the 14th position to tryptophan (W) in EgERF113 changes the binding specificity from GCC-box only to binding both GCC-box and DRE/CRT motifs, as suggested by Phukan et al. [39]. An additional amino acid (basic polar) at the 24th position of DBD which is lacking in EgERF113 is essential for specific binding to GCC-box only. Thus, EgERF113 with 60 amino acids DBD opposes the classification of specific GCC-box binding DBD of 61 amino acids in length.

### Proposed defense-related pathways regulated by *EgERF113* co-expressing genes

Two *RBOH* genes, *EgRBOHA* and *EgRBOHB* have been previously reported in our study [5], and reduced *EgRBOHB* expression during the early necrotrophic phase may suggest the plant’s response in delaying the progression of the disease. This is in line with a report on the response against hemibiotroph *Macrophomina phaseolina* [85], wherein increased expression of *RBOH* leads to increase susceptibility of plants to necrotrophic infection.

Another less studied TF, *AtMYB122* was reported to regulate genes involved in camalexin biosynthesis [86]. Increased accumulation of indolic glucosinolates during induction of glucose signaling was regulated by the *AtMYB122* [87]. In another similar reports, the glucosinolates and their derivatives contribute in defense resistance against necrotrophic fungi [88, 89]. Based on the expression patterns of *EgMYB122*, the TF channeled its regulation from responding to abiotic stress at 3 d.p.i. into defensing against *Ganoderma* attack and it is expected that the gene regulation will be more extensive at a later stage of the necrotrophic phase.

Rapid and transient increase of cytosolic Ca^2+^ particularly during pathogens interaction results in activation of both PTI and ETI signaling [84]. CMLs and CDPKs are known as Ca^2+^ sensor proteins which are responsible for perceiving the transduction during plant innate immunity [90, 91]. Based on our RNA-seq data analysis, both *EgCML7* and *EgCDPK28* were upregulated during the necrotrophic phase. Consistent with the analysis, EgERF113 is suggested to orchestrate defense mechanisms through the regulation of *PR* and calcium-responsive genes.

### Other transcription factors differentially regulated under the biotrophic or necrotrophic phase

The *Calmodulin-binding transcription activator* (*CAMTA*) gene was first discovered in *Nicotiana tabacum*, regulating senescence and cell death [92], and was recently comprehensively studied by Kakar et al. [93]. We discovered members of the novel *CAMTA* TF family, namely *EgCAMTA3* and *EgCAMTA4* which were downregulated at 3 d.p.i. The suppression of both genes was later reduced across time, which might be the result of infection phase transition from biotrophic to early necrotrophic phase. *CAMTA* was proven to regulate responses under Ca^2+^ signaling during both abiotic and biotic stresses [94, 95].

In general, most downregulated DEGs of TFs showed de-repression across time. It is reasonable to postulate that changes of expression patterns might be the results of plant immunity interplay against biotrophic and necrotrophic infection phases. For instance, downregulation of *EgMYB108* was highest at 7 d.p.i before reducing at 11 d.p.i. Coherently, *MYB108* was proven to positively regulate the defense mechanism against hemibiotroph *Verticillium dahliae* in the presence of calmodulin and Ca^2^, antagonistic to the regulation of *CAMTA3* [96, 97]. In contrast, *EgERF9* was found to be downregulated exclusively during the necrotrophic phase at 11 d.p.i. The result provides agreement with other studies which reported repression activity of *AtERF9* in enhanced resistance against necrotrophic *Botrytis cinerea* [82]. Likewise, expression patterns of TFs in upregulated DEGs were higher at early interaction against *G. boninense* before decreasing over time. The plant-specific *EgTCP15* demonstrated upregulation during the biotrophic phase before declining but showing an opposite expression pattern under abiotic stress.

## Conclusions

**Fig. 6:**
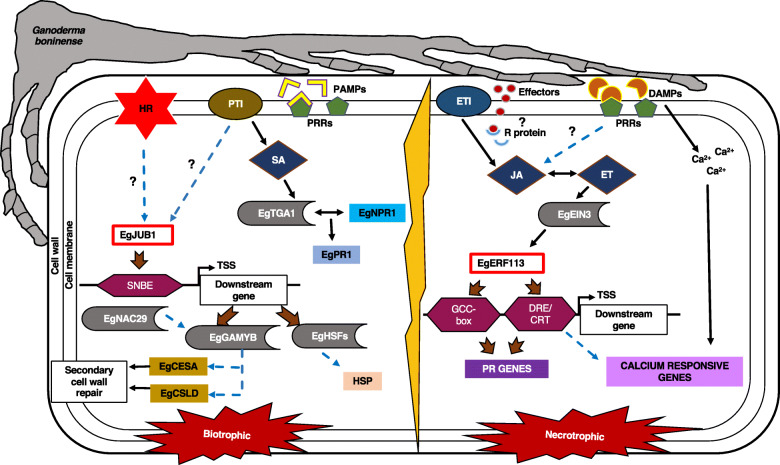
Proposed defense mechanisms of oil palm seedlings against hemibiotroph *Ganoderma boninense* during biotrophic and necrotrophic infection phases. Based on the NGS data on differentially expressed genes encoding TFs, two oil palm transcription factors (TFs), *EgJUB1* and *EgERF113* were discovered to regulate specifically under biotic stress during biotrophic and necrotrophic phases, respectively. The EgJUB1 TF binds to SNBE motif and directly regulates EgGAMYB and EgHSFs. The EgERF113 TF binds to GCC-box and DRE/CRT motifs which promotes *PR* and calcium responsive genes. The oil palm defense pathways are identified based on established defense mechanisms and new findings from this study. Black arrows represent direct regulation by encoded proteins/genes; blue arrows with broken lines suggest highly probable defense regulatory pathways; brown arrows indicate activation of downstream targets through binding to respective motifs. The question marks highlight the gaps in the oil palm defense mechanism that need to be further elucidated. Ca: calcium; CESA: cellulose synthase; CSLD: cellulose synthase-like D; DAMPs: damage-associated molecular patterns; DRE/CRT: dehydration response element/C-repeat; EIN3: ethylene insensitive 3; ET: ethylene; ETI: effector-triggered immunity; HR: hypersensitive response; HSFs: heat stress transcription factors; HSP: heat shock protein; JA: jasmonic acid; NPR1: NONEXPRESSOR OF PATHOGENESIS-RELATED GENE 1; PR: pathogenesis-related; PRRs: pattern recognition receptors; PTI: PAMP-triggered immunity; secondary wall NAC binding element (SNBE); SA: salicylic acid; TSS: transcription start site

Together with the results presented above, we were able to recognize TF genes that were regulated during switching of the fungal mode of infection. With this first analysis of oil palm RNA-seq data encoding TFs, six common major families of TFs were identified to be responsible for promoting oil palm defense response against *G. boninense* attack which include MYB, bHLH, AP2/ERF, NAC, bZIP and WRKY. Reported genes involved in cell wall modification, ROS-mediated signaling, PCD and plant innate immunity were all differentially expressed; indicating active regulation of oil palm defense response against the hemibiotrophic *G. boninense*. The biotrophic and necrotrophic infection phases of *G. boninense* were further supported through gene expression of biotrophy-specific, *EgJUB1* and necrotrophy-specific, *EgERF113*. Our finding is the first reporting EgJUB1 as a potential master regulator based on its positive interaction with the imperfect palindromic SNBE consensus sequence which may promote branches of biotrophy-associated defense mechanisms including cell wall strengthening and HR-mediated defense responses. Besides, EgERF113 is the first AP2/ERF TF reported to modulate multifaceted defense mechanisms through binding to GCC-box and DRE/CRT motifs during the necrotrophic phase. Binding to these motifs may result in transcriptional upregulation of *PR* and calcium-responsive genes. Based on our findings, a proposed defense mechanism inferring oil palm against hemibiotroph *G. boninense* during biotrophic and necrotrophic infection phases is illustrated as in Fig. 6. The information presents a promising first step in recognizing the downstream target defense-related genes regulated by the infection phase-specific TFs. Although our present study proposes important insights into the defense roles of EgJUB1 and EgERF113, over-expression studies or mutant complementation studies utilizing plant models such as *Arabidopsis thaliana* or *Nicotiana* spp. should be carried out in future investigations to further delineate defense mechanisms triggered by these TFs.

## Methods

### Plant materials and fungal treatment

Four-months old germinated oil palm seedlings Commercial DxP GH500 series (*Elaeis guineensis* Jacq. *Dura x Pisifera*), were purchased from Sime Darby Seeds and Agriculture Services Sdn. Bhd., Banting, Selangor, Malaysia. Pathogenic *Ganoderma boninense* strain PER71 was isolated and purified from an infected oil palm in United Plantation Teluk Intan, Perak, Malaysia [5], obtained from GanoDROP Unit, Biology Division, Malaysian Palm Oil Board (MPOB). Artificial infection of oil palm seedlings with *G. boninense* using rubber wood blocks (RWBs) was carried out following a previous study [5]. Control (C) was set as seedlings without treatment. Two different treatments were carried out; mock treatment (MT) consisted of oil palm seedlings with bare RWBs while *Ganoderma* treatment (GT) involved oil palm seedling treated with *Ganoderma*-inoculated RWBs. Destructive sampling was performed on two pooled biological replicates of oil palm seedlings at different days post-inoculation (3, 7 and 11 d.p.i). Each biological replicate consisted of pooled RNA provided equally from six constituent seedlings. Instead of mathematical averaging of individual samples, biological averaging is more cost-efficient and commonly practiced in the attempt of reducing high biological variability among samples in RNA-seq studies [98]. Pooling bias can be reduced by using three to eight biological individual samples per pool with two pools per treatment group [99].

### RNA extraction and DEGs analysis of TFs

Total RNA of all samples was extracted following the method reported in Bahari et al. [5]. The extracted RNA was used in all subsequent experiments. A high-throughput NGS data analysis was performed as described in our previous report, Bahari et al. [5]. The mRNA fragments were mapped to *Elaeis guineensis* coding sequences as reference genome (retrieved from https://www.ebi.ac.uk/genomes/) through Geneious software version 9.1.5 (Biomatters Ltd.). From align/assemble tools, Geneious for RNA-Seq was used as mapper with medium-low sensitivity using clean reads before mapping. Upon completion of the mapping step, the transcript abundance of each sample was calculated as transcript per kilobase million (TPM). Sequences that were reproducible in both pooled biological replicates were chosen to eliminate biased profiling of transcripts due to manipulations stages during library construction [100]. Alteration of gene expression profile was analyzed by comparing genes expressed from control with GT samples. DEGs were further evaluated following stringent cut-off values of log_2_ FC ≥ |1.0| (corresponding to 2-fold or more upregulation/downregulation) and *P*-value < 0.01 [5, 101]. Comparative analysis was conducted between two biological replicates of control and GT samples at all time points. DEGs of TFs that met the cut-off values were clustered according to upregulated and downregulated genes. Transcripts that were identified in both pooled biological replicates were further analyzed for identification of DEGs. DEGs of a few stress-related genes involving cell wall modification, ROS production and PCD were mined from NGS data analysis.

### Validation by quantitative real-time PCR (qPCR)

qPCR was performed using qPCR Green Master Mix LRox (2X) (Biotechrabbit GmbH, Germany). Stability of five endogenous controls; *EgGAPDH2, EgNADH5, EgMSD, EgUBQ,* and *Egß-actin* were tested across treated and control samples. The qPCR analysis was performed using Bio-Rad CFX Manager™ Software version 3.1. Expression levels of all target genes were normalized with the expression level of the three most stable reference genes which were *EgGAPDH2, EgNADH5* and *Egß-actin*. Real-time PCR was carried out on control (C), mock-treated (MT) and *Ganoderma-*treated (GT) oil palm root samples at 3, 7 and 11 d.p.i. The data included three biological replicates of root samples of oil palm seedlings. Comparative analysis of expression levels was expressed as fold change ± standard error of mean (SEM) of three individual technical replicates at *P*-value < 0.01. Significant differences of expression levels between test groups to control were determined using one-way analysis of variance (ANOVA) followed by Tukey’s test for comparison between treatments. The primers used for the qPCR are listed in Table 1.

**Table 1:** List of primers for quantitative Real-Time PCR (qPCR)

Target Gene	Sense sequence (5′-3′)	Anti-sense sequence (5′-3′)
*EgJUB1*	AATGGAACTCAGTACCTCAGGC	ATTATCCTTCCAAGCTCATCCC
*EgERF113*	AGCAGCACTAAAGTTCAAAGG	GAATAAGGTCTGGGTAGGAGG
*EgTCP15*	GACAAACCCTAACAGCCAAAGTA	AAATGTAGCCCACTAGACATGGA
*EgEIN3*	GGAAGGAGAAGGTGAAGTTTGAT	CCATAAGGCTATGCTGAATTTTG
*EgMYB122*	AGTACCAGACAAGCTTGAAGGC	TTACATCCTTAGCTAAACGGGG
*Egß-actin*	GAGAGAGCGTGCTACTCATCTT	CGGAAGTGCTTCTGAGATCC
*EgNADH5*	GCTCCCCTTTATTTGAATACCC	AATAGTTAGAGATGCCGCAAGC
*EgGAPDH2*	GAAGGTCATCATATCTGCTCCC	CATCAACAGTCTTCTGAGTGGC

### Sequence characterization of EgJUB1 and EgERF113

Sequence analysis was carried out using Basic Local Alignment Search Tool (BLAST 2.9) accessed from http://www.ncbi.nlm.nih.gov. BLAST. A homology search was carried out using BLASTX algorithm by comparing the translated protein sequence of interest (EgJUB1 and EgERF113) with other protein sequences available in the National Centre of Biotechnology Information (NCBI) database. The nucleotide sequences were translated into protein sequences through publicly-accessible website, ExPASy Molecular Biology Server (http://web.expasy.org/translate/). Multiple sequence alignment of protein sequences from different species were carried out using T-Coffee software version 11.0 (http://tcoffee.crg.cat/apps/tcoffee/do:expresso). NLS was predicted using an accessible website of NLS Mapper (http://nls-mapper.iab.keio.ac.jp/cgi-bin/NLS_Mapper_form.cgi).

### Yeast one-hybrid (Y1H) assay

Coding regions of *EgJUB1* and *EgERF113* (500 ng total RNA) flanking SMART sequences were amplified from GT samples at 3 and 11 d.p.i, respectively, using Q5® Hot Start High-Fidelity 2x Master Mix (New England Biolabs). Purified SMART-*EgJUB1* and *-EgERF113* were further amplified by long-distance PCR using Advantage® 2 PCR Mix (Takara Bio). Putative tandem repeats of target baits fragments (NACBS, SNBE1, SNBE2, GCC-box and DRE/CRT) as well as the respective mutated fragments were cloned into pAbAi vector and integrated into the yeast genome. Minimal inhibitory concentration (MIC) of Aureobasidin A (AbA) for each bait- and mutant-reporter yeast strain was determined.

Yeast One-Hybrid screening was conducted using Yeastmaker™ Yeast Transformation System 2 (Takara Bio). Components for yeast co-transformation reaction were added in given orders as follows; 2 μg of SMART-*EgJUB1* or *-EgERF113*, 1 μg of pGAD7-Rec AD (SmaI-linearized), 50 μg of denatured yeastmaker carrier DNA, 50 μL of competent yeast cells Y1HGold [pAbAi-baits] or Y1HGold [pAbAi-mutants] and 500 μL of PEG/LiAc solution. Transformations were plated on selective plates SD/−Leu and SD/−Leu/AbA. Transformation control, p53 was plated on SD/−Leu/AbA^200^. Plates were incubated at 30 °C for 3 days. Confirmation of positive clones was performed by colony PCR using Matchmaker Insert Check PCR Mix 2 (Takara Bio) and sent for sequencing using T7 promoter primer. A single colony of positive clone was cultured in synthetic dropout (SD) medium lacking leucine (SD/−Leu) broth to an optical density (OD_600_) of 0.1 and diluted in 10-fold dilution series. From each dilution, 10 μL of yeast culture was spotted on SD/−Leu and SD/−Leu/AbA plates. Oligonucleotides and primers used in the Y1H assay are listed in Table 2.

**Table 2:** List of oligonucleotides for Yeast One-Hybrid (Y1H) assay and electrophoretic mobility shift assay (EMSA)

Target Motifs	DNA element sequences (5′ - 3′)
NACBS	**GATGCCGT**GATGCCGTGATGCCGTGATGCCGT
mNACBS	**GATGACGT**GATGACGTGATGACGTGATGACGT
SNBE1	**TTGTGTGCTGTGGAAGTTT**TTGTGTGCTGTGGAAGTTT
mSNBE1	**TTGTGGGCTGTGGAAGTTT**TTGTGGGCTGTGGAAGTTT
SNBE2	**TGGCTTGTGCAAAAAGTAA**TGGCTTGTGCAAAAAGTAA
mSNBE2	**TGGCTCGTGCAAAAAGTAA**TGGCTCGTGCAAAAAGTAA
GCC-box	**TAAGAGCCGCC**TAAGAGCCGCCTAAGAGCCGCCTAAGAGCCGCC
mGCC-box	**TAAGATCCTCC**TAAGATCCTCCTAAGATCCTCCTAAGATCCTCC
DRE/CRT	**TGCCGACAT**TGCCGACATTGCCGACATTGCCGACAT
mDRE/CRT	**TATTTACAT**TATTTACATTATTTACATTATTTACAT

The tandem repeats of DNA elements of each target are bold and underlined. Point mutations are bold and underlined with red

### Isolation of EgJUB1 and EgERF113 nuclear protein

A single colony of positive clones from the Y1H assay was incubated in 5 mL SD/−Leu broth with overnight shaking at 30 °C. Yeast culture was transferred into 45 mL SD/−Leu broth and further grown with overnight shaking (18–20 h) at 30 °C until OD_600_ reached 0.8–1.0. Yeast cells were pelleted by centrifugation at 1000 *g* for 5 mins at 4 °C. Pellet was washed with 30 mL ice-cold sterile ultrapure water and centrifuged at 1000 *g* for 5 mins at 4 °C. Pellet was immediately flash-frozen in liquid nitrogen and ground into a fine powder. Extraction of nuclear protein was carried out using NE-PER™ Nuclear and Cytoplasmic Extraction Reagents (Thermo Scientific). EgJUB1 and EgERF113 nuclear extracts were stored at − 80 °C.

### Electrophoretic mobility shift assay (EMSA)

Double-stranded of sense and anti-sense oligonucleotides were biotin-labelled using Biotin 3′ End DNA Labeling kit (Thermo Scientific). Oligonucleotides used are listed in Table 2. The binding reaction system of EMSA was prepared using LightShift EMSA Optimization and Control kit (Thermo Scientific). The binding mixture was resolved in 6% non-denaturing polyacrylamide gel in 0.5X Tris-borate-EDTA (TBE) buffer and transferred to a positively charged nylon membrane. The membrane was cross-linked at 120 mJ/cm^2^ for 1 min. The protein-DNA complexes were visualized by LightShift® Chemiluminescent EMSA kit (Thermo Scientific) according to the manufacturer’s protocol.

### Subcellular localization

The open reading frames of *EgJUB1* and *EgERF113* lacking stop codon were amplified using KAPA HiFi HotStart ReadyMix PCR kit (Thermo Scientific). The gene-specific primers with CACC flanking at 5’end of forward primer are listed in Table 3. The PCR products were transferred into the gateway pENTR/D-TOPO entry vector using the pENTR™ Directional Topo Cloning Kit (Thermo Fisher Scientific). TOPO Cloning Reaction was transformed into One Shot® TOP10 Chemically Competent cells (Thermo Fisher Scientific) to generate entry clone and sequence verified. The expression clones for EgJUB1 and EgERF113 were generated using Gateway LR Clonase II Enzyme Mix (Thermo Fisher Scientific) by recombination of entry clones into Gateway-compatible destination vector consisting double CaMV 35S promoter (2 x 35S) of pMDC85, respectively. The construction of pMDC85 vector without *ccd*B gene and insert was used as a negative control. The LR reaction was transformed into One Shot™ OmniMAX™ 2 T1® Chemically Competent cells (Thermo Fisher Scientific).

**Table 3:** List of primers for vector construction of subcellular localization

Target Gene	Sense sequence (5′-3′)	Anti-sense sequence (5′-3′)
*EgJUB1*	CACCATGGAGGAGAAGATGGACAA	AGCGTATCTACATTCATGACCGG
*EgERF113*	CACCATGGAGACCGAGATTAGAATCC	CTCTCTTGGTTGGCTAGTTTCTG

The resulting plasmids of 2x35S:GFP (negative control), 2x35S::EgJUB1-GFP and 2x35S::EgERF113-GFP were sequence-verified and transformed into competent *Agrobacterium tumefaciens* strain LBA4404 [102]. *Agrobacterium*-mediated transformation of onion epidermal cells was carried out according to Azzeme et al. [103]. Fresh onion scales (1.5 × 1 cm) were immersed into 20 mL *Agrobacterium* suspension harbouring control and GFP constructs, respectively for 16 h at 28 °C. The onion scales were transferred to a Murashige and Skoog (MS) medium pH 5.8 and further co-cultivated with *Agrobacterium* for 2 days. The peeled onion epidermal cells were rinsed with sterile water and transferred to glass slides. Fluorescence images were captured using a 20X lens of confocal laser scanning microscope (LSM 5 PASCAL EXCITER, Zeiss, Germany) with excitation at 488 nm and analyzed by LSM 5 Image Browser software (Ver. 4.1).

### Statistical analysis

For RNA-seq data analysis, DEGs were determined following cut off-values of log_2_ FC ≥ |1.0| and *P*-value < 0.01. Expression levels of each gene from qPCR analysis were normalized by three reference genes; *EgGAPDH2*, *EgNADH5* and *Egß-actin*. Data was presented as mean ± standard error of mean (SEM) of three independent technical replicates. Differences of expression level between samples at different time points to control and between group of treatments (MT and GT) were determined by using one-way ANOVA analysis followed by Tukey’s test. Significantly different expression levels as compared to the control were measured according to **P < 0.01, ***P < 0.001 and ****P < 0.0001. ns is defined as not significant. All graphs were generated and analyzed using GraphPad Prism version 5.0 (GraphPadSoftware Inc., USA).

## Supplementary Information

**Additional file 1:.** Electrophoretic mobility shift assay (EMSA) of EgJUB1 with SNBE1 probe. Lane 1 to 3 consist of EBNA control system. Lane 4 to 8 consist of EgJUB1 test system. Lane 1 and 8 are the blank for EBNA and EgJUB1 systems, respectively. Lane 2 is the positive control for EMSA. EMSA shows direct binding of EgJUB1 to SNBE1 probe in lane 6. EgJUB1 is unable to bind to untransformed yeast and biotinylated mutant SNBE1 (mSNBE1) probe in lane 4 and 5, respectively. Successful binding of 200-fold molar excess of unlabelled SNBE1 (competitor) probe is shown in lane 7.

**Additional file 2:.** Electrophoretic mobility shift assay (EMSA) of EgERF113 with GCC-box probe. Lane 1 to 3 consist of EBNA control system. Lanes 4 to 8 consist of EgERF113 test system. Lane 1 and 8 are the blank for EBNA and EgERF113 systems, respectively. Lane 2 is the positive control for EMSA. EMSA shows direct binding of EgERF113 to GCC-box probe in lane 6. EgERF113 is unable to bind to untransformed yeast and biotinylated mutant GCC-box (mGCC-box) probe in lane 4 and 5, respectively. Successful binding of 200-fold molar excess of unlabelled GCC-box (competitor) probe is shown in lane 7.

**Additional file 3:.** Electrophoretic mobility shift assay (EMSA) of EgERF113 with DRE/CRT probe. Lane 1 to 3 consist of EBNA control system. Lane 4 to 8 consist of EgERF113 test system. Lane 1 and 8 are the blank for EBNA and EgERF113 systems, respectively. Lane 2 is the positive control for EMSA. EMSA shows direct binding of EgERF113 to DRE/CRT probe in lane 6. EgERF113 is unable to bind to untransformed yeast and biotinylated mutant DRE/CRT (mDRE/CRT) probe in lane 4 and 5, respectively. Successful binding of 200-fold molar excess of unlabelled DRE/CRT (competitor) probe is shown in lane 7.

## Data Availability

The sequenced mRNA data was deposited at European Nucleotide Archive with accession number PRJEB27915. https://www.ebi.ac.uk/ena/data/view/PRJEB27915

## Abbreviations

A: Alanine
AbA: Aureobasidin A
ABA: Abscisic acid
ANOVA: Analysis of variance
AP2/ERF: APETALA2/ethylene responsive factor
Avr: Virulence effectors
bHLH: basic helix-loop-helix
BLAST: Basic Local Alignment Search Tool
bZIP: basic leucine zipper
Ca^2+^: Calcium ion
CAMTA: Calmodulin-binding transcription activator
BSR: Basal stem rot
CWDE: Cell wall degrading enzyme
DAMP: Damage-associated molecular pattern
DBD: DNA-binding domain
DEG: Differentially expressed gene
d.p.i: Days post inoculation
DREB: Dehydration responsive element-binding
DRE/CRT: Dehydration-Responsive Element/C-Repeat
EDTA: Ethylenediaminetetraacetic acid
EIN3: Ethylene-insensitive 3
EIL: EIN3-like
EMSA: Electrophoretic Mobility Shift Assay
ERE: Ethylene-Response Element
ERF: Ethylene Responsive Factor
ET: Ethylene
ETI: Effector-triggered immunity
F: Phenylalanine
G: Glutamic acid
GAPDH: Glyceraldehyde 3-phosphate dehydrogenase
GT: *Ganoderma*-treated
HR: Hypersensitive response
HSF: Heat stress transcription factor
HSP: Heat shock protein
Ile: Isoleucine
JA: Jasmonic acid
JAZ: Jasmonic acid-ZIM domain
JUB1: JUNGBRUNNEN 1
Leu: Leucine
LiAc: Lithium acetate
MIC: Minimal Inhibitory Concentration
MPOB: Malaysian Palm Oil Board
MT: Mock-treated
MYB: Myeloblastosis
MYC: Myelocytomatosis
NADH: Nicotinamide adenine dinucleotide dehydrogenase
NACBS: NAC binding site
NCBI: National Centre of Biotechnology Information
NGS: Next-Generation Sequencing
NLS: Nuclear localization signals
NPR1: NONEXPRESSOR OF PATHOGENESIS-RELATED GENE 1
ns: Not significant
OD: Optical density
PAMP: Pathogen-associated molecular pattern
PlantTFDB: Plant Transcription Factor Database
PCD: Programmed cell death
PR: Pathogenesis-related
PRR: Pattern recognition receptor
PTI: PAMP-triggered immunity
qPCR: Quantitative Real-Time Polymerase Chain Reaction
RAP2.6: RELATED TO APETALA 2.6
RAP2.6 L: RELATED TO APETALA 2.6-LIKE
RBOH: Respiratory burst oxidase homolog protein
ROS: Reactive Oxygen Species
RT: Room temperature
RWB: Rubber wood block
SA: Salicylic acid
SD: Synthetic dropout
SEM: Standard error of mean
SNBE: Secondary wall NAC binding element
SOD: Superoxide dismutase
TBE: Tris-borate-EDTA
TF: Transcription factor
TPM: Transcript per kilobase million
TSS: Transcription start site
W: Tryptophan
Y1H: Yeast One-Hybrid

## References

[1] Rees RW, Flood J, Hasan Y, Potter U, Cooper RM (2009). Basal stem rot of oil palm (*Elaeis guineensis*); mode of root infection and lower stem invasion by *Ganoderma boninense*. Plant Pathol.

[2] Mohammed CL, Rimbawanto A, Page DE (2014). Management of basidiomycete root-and stem-rot diseases in oil palm, rubber and tropical hardwood plantation crops. Forest Pathol.

[3] Chong KP, Dayou J, Alexander A (2017). Pathogenic nature of *Ganoderma boninense* and basal stem rot disease. Detection and control of *Ganoderma boninense* in oil palm crop.

[4] Ho CL, Tan YC (2014). Molecular defense response of oil palm to *Ganoderma* infection. Phytochemistry.

[5] Bahari MNA, Sakeh NM, Abdullah SNA, Ramli RR, Kadkhodaei S (2018). Transciptome profiling at early infection of *Elaeis guineensis* by *Ganoderma boninense* provides novel insights on fungal transition from biotrophic to necrotrophic phase. BMC Plant Biol.

[6] Dodds PN, Rafiqi M, Gan PH, Hardham AR, Jones DA, Ellis JG (2009). Effectors of biotrophic fungi and oomycetes: pathogenicity factors and triggers of host resistance. New Phytol.

[7] Catanzariti AM, Dodds PN, Ellis JG (2007). Avirulence proteins from haustoria-forming pathogens. FEMS Microbiol Lett.

[8] Zhao Z, Liu H, Wang C, Xu JR (2014). Erratum to: comparative analysis of fungal genomes reveals different plant cell wall degrading capacity in fungi. BMC Genomics.

[9] Nusaibah SA, Abdullah SNA, Idris AS, Sariah M, Pauzi ZM (2016). Involvement of metabolites in early defense mechanism of oil palm (*Elaeis guineensis* Jacq.) against *Ganoderma* disease. Plant Physiol Biochem.

[10] Boller T, Felix G (2009). A renaissance of elicitors: perception of microbe-associated molecular patterns and danger signals by pattern-recognition receptors. Annu Rev Plant Biol.

[11] Dodds PN, Rathjen JP (2010). Plant immunity: towards an integrated view of plant–pathogen interactions. Nat Rev Genet.

[12] Cook DE, Mesarich CH, Thomma BP (2015). Understanding plant immunity as a surveillance system to detect invasion. Annu Rev Phytopathol.

[13] Pel MJ, Pieterse CM (2012). Microbial recognition and evasion of host immunity. J Exp Bot.

[14] Zipfel C (2014). Plant pattern-recognition receptors. Trends Immunol.

[15] Miller RNG, Costa Alves GS, Van Sluys MA (2017). Plant immunity: unravelling the complexity of plant responses to biotic stresses. Ann Bot.

[16] Yi M, Valent B (2013). Communication between filamentous pathogens and plants at the biotrophic interface. Annu Rev Phytopathol.

[17] Rossi FR, Krapp AR, Bisaro F, Maiale SJ, Pieckenstain FL, Carrillo N (2017). Reactive oxygen species generated in chloroplasts contribute to tobacco leaf infection by the necrotrophic fungus *Botrytis cinerea*. Plant J.

[18] Vargas WA, Martín JMS, Rech GE, Rivera LP, Benito EP, Díaz-Mínguez JM, Thon MR, Sukno SA (2012). Plant defense mechanisms are activated during biotrophic and necrotrophic development of *Colletotricum* graminicola in maize. Plant Physiol.

[19] Kabbage M, Yarden O, Dickman MB (2015). Pathogenic attributes of *Sclerotinia sclerotiorum*: switching from a biotrophic to necrotrophic lifestyle. Plant Sci.

[20] Abdullah SNA, Akhtar MS (2016). Plant and necrotrophic fungal pathogen interaction: mechanism and mode of action. Plant, soil and microbes.

[21] Azizi P, Rafii MY, Abdullah SNA, Nejat N, Maziah M, Hanafi MM, Latif MA, Sahebi M (2016). Toward understanding of rice innate immunity against *Magnaporthe oryzae*. Crit Rev Biotechnol.

[22] Häffner E, Konietzki S, Diederichsen E (2015). Keeping control: the role of senescence and development in plant pathogenesis and defense. Plants.

[23] Giri MK, Singh N, Banday ZZ, Singh V, Ram H, Singh D, Chattopadhyay S, Nandi AK (2017). GBF 1 differentially regulates *CAT2* and *PAD4* transcription to promote pathogen defense in *Arabidopsis thaliana*. Plant J.

[24] Liu B, Ouyang Z, Zhang Y, Li X, Hong Y, Huang L, Liu S, Zhang H, Li D, Song F (2014). Tomato NAC transcription factor SlSRN1 positively regulates defense response against biotic stress but negatively regulates abiotic stress response. PLoS One.

[25] Rasmussen S, Barah P, Suarez-Rodriguez MC, Bressendorff S, Friis P, Costantino P, Bones AM, Nielsen HB, Mundy J (2013). Transcriptome responses to combinations of stresses in *Arabidopsis*. Plant Physiol.

[26] Ng DW, Abeysinghe JK, Kamali M (2018). Regulating the regulators: the control of transcription factors in plant defense signaling. Int J Mol Sci.

[27] Pandey D, Rajendran SRCK, Gaur M, Sajeesh PK, Kumar A (2016). Plant defense signaling and responses against necrotrophic fungal pathogens. J Plant Growth Regul.

[28] Foyer CH, Rasool B, Davey JW, Hancock RD (2016). Cross-tolerance to biotic and abiotic stresses in plants: a focus on resistance to aphid infestation. J Exp Bot.

[29] Guo M, Liu JH, Ma X, Luo DX, Gong ZH, Lu MH (2016). The plant heat stress transcription factors (HSFs): structure, regulation, and function in response to abiotic stresses. Front Plant Sci.

[30] Fragkostefanakis S, Simm S, El-Shershaby A, Hu Y, Bublak D, Mesihovic A, Darm K, Mishra SK, Tschiersch B, Theres K, Scharf C, Schleiff E, Scharf KD (2019). The repressor and co-activator HsfB1 regulates the major heat stress transcription factors in tomato. Plant Cell Environ.

[31] Bechtold U, Albihlal WS, Lawson T, Fryer MJ, Sparrow PAC, Richard F, Persad R, Bowden L, Hickman R, Martin C, Beynon JL, Buchanan-Wollaston V, Baker NR, Morison JIL, Schöffl F, Ott S, Mullineaux PM (2013). *Arabidopsis* HEAT SHOCK TRANSCRIPTION FACTORA1b overexpression enhances water productivity, resistance to drought, and infection. J Exp Bot.

[32] Ramli Z, Abdullah SNA (2010). Functional characterisation of the oil palm type 3 metallothionein-like gene (MT3-B) promoter. Plant Mol Biol Rep.

[33] Hernandez-Garcia CM, Finer JJ (2014). Identification and validation of promoters and cis-acting regulatory elements. Plant Sci.

[34] Sanchez I, Hernandez-Guerrero R, Mendez-Monroy PE, Martinez-Nuñez MA, Ibarra JA, Pérez-Rueda E (2020). Evaluation of the abundance of DNA-binding transcription factors in prokaryotes. Genes.

[35] Huang D, Wang S, Zhang B, Shang-Guan K, Shi Y, Zhang D, Liu X, Wu K, Xu Z, Fu X, Zhou YA (2015). Gibberellin-mediated DELLA-NAC signaling cascade regulates cellulose synthesis in rice. Plant Cell.

[36] Li S (2014). Transcriptional control of flavonoid biosynthesis: fine-tuning of the MYB-bHLH-WD40 (MBW) complex. Plant Signal Behav.

[37] Nemesio-Gorriz M, Blair PB, Dalman K, Hammerbacher A, Arnerup J, Stenlid J, Mukhtar SM, Elfstrand M (2017). Identification of Norway spruce MYB-bHLH-WDR transcription factor complex members linked to regulation of the flavonoid pathway. Front Plant Sci.

[38] Sun X, Malhis N, Zhao B, Xue B, Gsponer J, Rikkerink EH (2020). Computational disorder analysis in ethylene response factors uncovers binding motifs critical to their diverse functions. Int J Mol Sci.

[39] Phukan UJ, Jeena GS, Tripathi V, Shukla RK (2017). Regulation of Apetala2/ethylene response factors in plants. Front Plant Sci.

[40] Purohit A, Ganguly S, Chaudhuri RK, Chakraborti D. Understanding the Interaction of Molecular Factors During the Crosstalk Between Drought and Biotic Stresses in Plant. In: Molecular Plant Abiotic Stress: Biology and Biotechnology. Hoboken: Wiley; 2019. p. 427–46.

[41] Tee SS, Tan YC, Abdullah F, Ong-Abdullah M, Ho CL (2013). Transcriptome of oil palm (*Elaeis guineensis* Jacq.) roots treated with *Ganoderma boninense*. Tree Genet Genomes.

[42] Ho CL, Tan YC, Yeoh KA, Ghazali AK, Yee WY, Hoh CC (2016). De novo transcriptome analyses of host-fungal interactions in oil palm (*Elaeis guineensis* Jacq.). BMC Genomics.

[43] Yuan P, Du L, Poovaiah B (2018). Ca^2+/^Calmodulin-dependent AtSR1/CAMTA3 plays critical roles in balancing plant growth and immunity. Int J Mol Sci.

[44] Wang Y, Wei F, Zhou H, Liu N, Niu X, Yan C, Zhang L, Han S, Hou C, Wang D (2019). TaCAMTA4, a Calmodulin-interacting protein, involved in defense response of wheat to *Puccinia triticina*. Sci Rep.

[45] Viola IL, Camoirano A, Gonzalez DH (2016). Redox-dependent modulation of anthocyanin biosynthesis by the TCP transcription factor TCP15 during exposure to high light intensity conditions in *Arabidopsis*. Plant Physiol.

[46] Li M, Chen H, Chen J, Chang M, Palmer IA, Gassmann W, Liu F, Fu Z (2018). TCP transcription factors interact with NPR1 and contribute redundantly to systemic acquired resistance. Front Plant Sci.

[47] Zhang X, Ji Y, Xue C, Ma H, Xi Y, Huang P, Wang H, An F, Li B, Wang Y, Guo H (2018). Integrated regulation of apical hook development by transcriptional coupling of EIN3/EIL1 and PIFs in *Arabidopsis*. Plant Cell.

[48] Ramirez-Prado JS, Latrasse D, Rodriguez-Granados NY, Huang Y, Manza-Mianza D, Brik-Chaouche R, Jaouannet M, Citerne S, Bendahmane A, Hirt H, Raynaud C (2019). The Polycomb protein LHP1 regulates Arabidopsis thaliana stress responses through the repression of the MYC2-dependent branch of immunity. Plant J.

[49] Zhong R, Lee C, Ye ZH (2010). Global analysis of direct targets of secondary wall NAC master switches in *Arabidopsis*. Mol Plant.

[50] Laluk K, Mengiste T (2010). Necrotroph attacks on plants: wanton destruction or covert extortion?. Arabidopsis Book.

[51] Wu A, Allu AD, Garapati P, Siddiqui H, Dortay H, Zanor MI, Asensi-Fabado MA, Munné-Bosch S, Antonio C, Tohge T, Fernie AR (2012). JUNGBRUNNEN1, a reactive oxygen species–responsive NAC transcription factor, regulates longevity in *Arabidopsis*. Plant Cell.

[52] Ohama N, Sato H, Shinozaki K, Yamaguchi-Shinozaki K (2017). Transcriptional regulatory network of plant heat stress response. Trends Plant Sci.

[53] Saga H, Ogawa T, Kai K, Suzuki H, Ogata Y, Sakurai N, Shibata D, Ohta D (2012). Identification and characterization of ANAC042, a transcription factor family gene involved in the regulation of camalexin biosynthesis in *Arabidopsis*. Mol Plant-Microbe Interact.

[54] Toth Z, Winterhagen P, Kalapos B, Su Y, Kovacs L, Kiss E (2016). Expression of a grapevine NAC transcription factor gene is induced in response to powdery mildew colonization in salicylic acid-independent manner. Sci Rep.

[55] Lindermayr C, Sell S, Müller B, Leister D, Durner J (2010). Redox regulation of the NPR1-TGA1 system of *Arabidopsis thaliana* by nitric oxide. Plant Cell.

[56] Qi G, Chen J, Chang M, Chen H, Hall K, Korin J, Liu F, Wang D, Fu ZQ (2018). Pandemonium breaks out: disruption of salicylic acid-mediated defense by plant pathogens. Mol Plant.

[57] McCarthy RL, Zhong R, Ye Z (2011). Secondary wall NAC binding element (SNBE), a key cis-acting element required for target gene activation by secondary wall NAC master switches. Plant Signal Behav.

[58] Kallamadi PR, Dandu K, Kirti PB, Rao CM, Thakur SS, Mulpuri S (2018). An insight into powdery mildew–infected, susceptible, resistant, and immune sunflower genotypes. Proteomics.

[59] Neu E, Domes HS, Menz I, Kaufmann H, Linde M, Debener T (2019). Interaction of roses with a biotrophic and a hemibiotrophic leaf pathogen leads to differences in defense transcriptome activation. Plant Mol Biol.

[60] Wei Y, Liu G, Chang Y, He C, Shi H (2018). Heat shock transcription factor 3 regulates plant immune response through modulation of salicylic acid accumulation and signaling in cassava. Mol Plant Pathol.

[61] Woodger FJ, Millar A, Murray F, Jacobsen JV, Gubler F (2003). The role of GAMYB transcription factors in GA-regulated gene expression. J Plant Growth Regul.

[62] Wei K, Zhao Y, Zhou H, Jiang C, Zhang B, Zhou Y, Song X, Lu M (2019). PagMYB216 is involved in the regulation of cellulose synthesis in *Populus*. Mol Breed.

[63] Tanaka K, Murata K, Yamazaki M, Onosato K, Miyao A, Hirochika H (2003). Three distinct rice cellulose synthase catalytic subunit genes required for cellulose synthesis in the secondary wall. Plant Physiol.

[64] Després C, Chubak C, Rochon A, Clark R, Bethune T, Desveaux D, Fobert PR (2003). The *Arabidopsis* NPR1 disease resistance protein is a novel cofactor that confers redox regulation of DNA binding activity to the basic domain/leucine zipper transcription factor TGA1. Plant Cell.

[65] Ndamukong I, Abdallat AA, Thurow C, Fode B, Zander M, Weigel R, Gatz C (2007). SA-inducible *Arabidopsis* glutaredoxin interacts with TGA factors and suppresses JA-responsive PDF1. 2 transcription. Plant J.

[66] Wen X, Zhang C, Ji Y, Zhao Q, He W, An F, Jiang L, Guo H (2012). Activation of ethylene signaling is mediated by nuclear translocation of the cleaved EIN2 carboxyl terminus. Cell Res.

[67] Yoo SD, Cho Y, Sheen J (2009). Emerging connections in the ethylene signaling network. Trends Plant Sci.

[68] Nieuwenhuizen NJ, Chen X, Wang MY, Matich AJ, Perez RL, Allan AC, Green SA, Atkinson RG (2015). Natural variation in monoterpene synthesis in kiwifruit: transcriptional regulation of terpene synthases by NAC and ETHYLENE-INSENSITIVE3-like transcription factors. Plant Physiol.

[69] Zhu Z, An F, Feng Y, Li P, Xue L, Mu A, Jiang Z, Kim JM, Li W, Zhang X, To TK (2011). Derepression of ethylene-stabilized transcription factors (EIN3/EIL1) mediates jasmonate and ethylene signaling synergy in *Arabidopsis*. Proc Natl Acad Sci.

[70] Krishnaswamy S, Verma S, Rahman MH, Kav NN (2011). Functional characterization of four APETALA2-family genes (RAP2. 6, RAP2. 6L, DREB19 and DREB26) in *Arabidopsis*. Plant Mol Biol.

[71] Wang Z, Cao G, Wang X, Miao J, Liu X, Chen Z, Qu LJ, Gu H (2008). Identification and characterization of COI1-dependent transcription factor genes involved in JA-mediated response to wounding in *Arabidopsis* plants. Plant Cell Rep.

[72] Ali MA, Abbas A, Kreil DP, Bohlmann H (2013). Overexpression of the transcription factor RAP2. 6 leads to enhanced callose deposition in syncytia and enhanced resistance against the beet cyst nematode *Heterodera schachtii* in *Arabidopsis* roots. BMC Plant Biol.

[73] Asahina M, Azuma K, Pitaksaringkarn W, Yamazaki T, Mitsuda N, Ohme-Takagi M, Yamaguchi S, Kamiya Y, Okada K, Nishimura T, Koshiba T, Yokota T, Kamada H, Satoh S (2011). Spatially selective hormonal control of RAP2. 6L and ANAC071 transcription factors involved in tissue Reunion in *Arabidopsis*. Proc Natl Acad Sci.

[74] Matsuoka K, Yanagi R, Yumoto E, Yokota T, Yamane H, Satoh S, Asahina M (2018). RAP2. 6L and jasmonic acid–responsive genes are expressed upon *Arabidopsis* hypocotyl grafting but are not needed for cell proliferation related to healing. Plant Mol Biol.

[75] Wang H, Lin J, Chang Y, Jiang CZ (2017). Comparative transcriptomic analysis reveals that ethylene/H_2_O_2_-mediated hypersensitive response and programmed cell death determine the compatible interaction of sand pear and *Alternaria alternata*. Front Plant Sci.

[76] Zhao Y, Chang X, Qi D, Dong L, Wang G, Fan S, Jiang L, Cheng Q, Chen X, Han D, Xu P, Zhang S (2017). A novel soybean ERF transcription factor, GmERF113, increases resistance to *Phytophthora sojae* infection in soybean. Front Plant Sci.

[77] Franco-Zorrilla JM, López-Vidriero I, Carrasco JL, Godoy M, Vera P, Solano R (2014). DNA-binding specificities of plant transcription factors and their potential to define target genes. Proc Natl Acad Sci.

[78] Park JM, Park CJ, Lee SB, Ham BK, Shin R, Paek K (2001). Overexpression of the tobacco Tsi1 gene encoding an EREBP/AP2–type transcription factor enhances resistance against pathogen attack and osmotic stress in tobacco. Plant Cell.

[79] Lee JH, Hong JP, Oh SK, Lee S, Choi D, Kim W (2004). The ethylene-responsive factor like protein 1 (CaERFLP1) of hot pepper (*Capsicum annuum* L.) interacts *in vitro* with both GCC and DRE/CRT sequences with different binding affinities: possible biological roles of CaERFLP1 in response to pathogen infection and high salinity conditions in transgenic tobacco plants. Plant Mol Biol.

[80] Lee JH, Kim DM, Lee JH, Kim J, Bang JW, Kim WT, Pai HS (2005). Functional characterization of NtCEF1, an AP2/EREBP-type transcriptional activator highly expressed in tobacco callus. Planta.

[81] Zhang G, Chen M, Li L, Xu Z, Chen X, Guo J, Ma Y (2009). Overexpression of the soybean *GmERF3* gene, an AP2/ERF type transcription factor for increased tolerances to salt, drought, and diseases in transgenic tobacco. J Exp Bot.

[82] Maruyama Y, Yamoto N, Suzuki Y, Chiba Y, Yamazaki KI, Sato T, Yamaguchi J (2013). The *Arabidopsis* transcriptional repressor ERF9 participates in resistance against necrotrophic fungi. Plant Sci.

[83] Kaur A, Pati PK, Pati AM, Nagpal AK (2017). *In-silico* analysis of cis-acting regulatory elements of pathogenesis-related proteins of *Arabidopsis thaliana* and *Oryza sativa*. PLoS One.

[84] Aldon D, Mbengue M, Mazars C, Galaud JP (2018). Calcium signaling in plant biotic interactions. Int J Mol Sci.

[85] Chowdhury S, Basu A, Kundu S (2017). Biotrophy-necrotrophy switch in pathogen evoke differential response in resistant and susceptible sesame involving multiple signaling pathways at different phases. Sci Rep.

[86] Frerigmann H, Glawischnig E, Gigolashvili T (2015). The role of MYB34, MYB51 and MYB122 in the regulation of camalexin biosynthesis in *Arabidopsis thaliana*. Front Plant Sci.

[87] Miao H, Cai C, Wei J, Huang J, Chang J, Qian H, Zhang X, Zhao Y, Sun B, Wang B, Wang Q (2016). Glucose enhances indolic glucosinolate biosynthesis without reducing primary sulfur assimilation. Sci Rep.

[88] Kos M, Houshyani B, Achhami BB, Wietsma R, Gols R, Weldegergis BT, Kabouw P, Bouwmeester HJ, Vet LE, Dicke M, van Loon JJ (2012). Herbivore-mediated effects of glucosinolates on different natural enemies of a specialist aphid. J Chem Ecol.

[89] Buxdorf K, Yaffe H, Barda O, Levy M (2013). The effects of glucosinolates and their breakdown products on necrotrophic fungi. PLoS One.

[90] Cheval C, Aldon D, Galaud JP, Ranty B (2013). Calcium/calmodulin-mediated regulation of plant immunity. BBA Mol Cell Res.

[91] Gao X (2014). Cox JrK, He P. functions of calcium-dependent protein kinases in plant innate immunity. Plants.

[92] Yang T, Poovaiah BW (2000). An early ethylene up-regulated gene encoding a calmodulin-binding protein involved in plant senescence and death. J Biol Chem.

[93] Kakar KU, Nawaz Z, Cui Z, Cao P, Jin J, Shu Q, Ren X (2018). Evolutionary and expression analysis of CAMTA gene family in *Nicotiana tabacum* yielded insights into their origin, expansion and stress responses. Sci Rep.

[94] Yue R, Lu C, Sun T, Peng T, Han X, Qi J, Yan S, Tie S (2015). Identification and expression profiling analysis of calmodulin-binding transcription activator genes in maize (*Zea mays* L.) under abiotic and biotic stresses. Front. Plant Sci.

[95] Kollist H, Zandalinas SI, Sengupta S, Nuhkat M, Kangasjärvi J, Mittler R (2019). Rapid responses to abiotic stress: priming the landscape for the signal transduction network. Trends Plant Sci.

[96] Zhang L, Du L, Shen C, Yang Y, Poovaiah BW (2014). Regulation of plant immunity through ubiquitin-mediated modulation of Ca^2+^–calmodulin–AtSR 1/CAMTA 3 signaling. Plant J.

[97] Cheng HQ, Han LB, Yang CL, Wu XM, Zhong NQ, Wu JH, Wang FX, Wang HY (2016). Xia, G. X. the cotton MYB108 forms a positive feedback regulation loop with CML11 and participates in the defense response against *Verticillium dahliae* infection. J Exp Bot.

[98] Kendziorski C, Irizarry RA, Chen KS, Haag JD, Gould MN (2005). On the utility of pooling biological samples in microarray experiments. Proc Natl Acad Sci.

[99] Rajkumar AP, Qvist P, Lazarus R, Lescai F, Ju J, Nyegaard M, Mors O, Borglum AD, Li Q, Christensen JH (2015). Experimental validation of methods for differential gene expression analysis and sample pooling in RNA-seq. BMC Genomics.

[100] Wang Z, Gerstein M, Snyder M (2009). RNA-Seq: a revolutionary tool for transcriptomics. Nat Rev Genet.

[101] Han Y, Wan H, Cheng T, Wang J, Yang W, Pan H, Zhang Q (2017). Comparative RNA-seq analysis of transcriptome dynamics during petal development in *Rosa chinensis*. Sci Rep.

[102] Höfgen R, Willmitzer L (1988). Storage of competent cells for *Agrobacterium* transformation. Nucleic Acids Res.

[103] Azzeme AM, Abdullah SNA, Aziz MA, Wahab PEM (2017). Oil palm drought inducible DREB1 induced expression of DRE/CRT-and non-DRE/CRT-containing genes in lowland transgenic tomato under cold and PEG treatments. Plant Physiol Biochem.

