# Supplementary material for: EgJUB1 and EgERF113 transcription factors as potential master regulators of defense response in Elaeis guineensis against the hemibiotrophic Ganoderma boninense
Source: BMC Plant Biol. 2021 Jan 22;21:59. doi: 10.1186/s12870-020-02812-7 (PMC7825162; doi:10.1186/s12870-020-02812-7)

**Additional file 1 :** **Electrophoretic mobility shift assay (EMSA) of EgJUB1 with SNBE1 probe.** Lane 1 to 3 consist of EBNA control system. Lane 4 to 8 consist of EgJUB1 test system. Lane 1 and 8 are the blank for EBNA and EgJUB1 systems, respectively. Lane 2 is the positive control for EMSA. EMSA shows direct binding of EgJUB1 to SNBE1 probe in lane 6. EgJUB1 is unable to bind to untransformed yeast and biotinylated mutant SNBE1 (mSNBE1) probe in lane 4 and 5, respectively. Successful binding of 200-fold molar excess of unlabelled SNBE1 (competitor) probe is shown in lane 7.

| **1** | **2** | **3** | **4** | **5** | **6** | **7** | **8** |
| --- | --- | --- | --- | --- | --- | --- | --- |


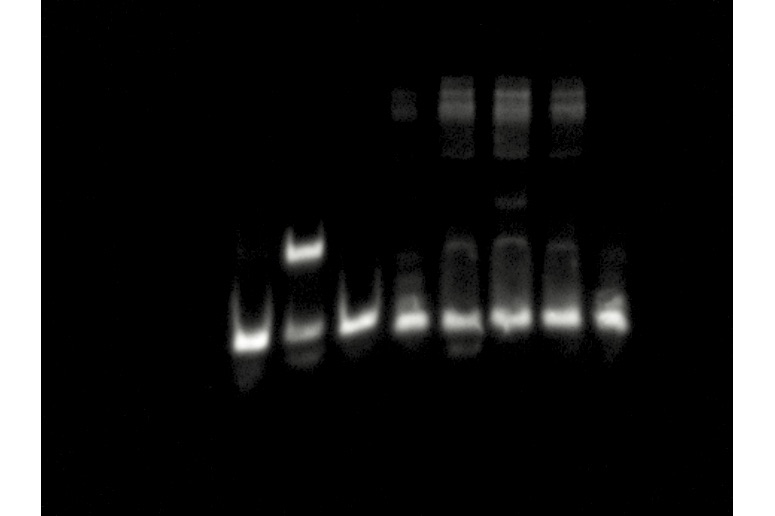

Supplement: Supplementary file 1 — Additional file 1:. Electrophoretic mobility shift assay (EMSA) of EgJUB1 with SNBE1 probe. Lane 1 to 3 consist of EBNA control system. Lane 4 to 8 consist of EgJUB1 test system. Lane 1 and 8 are the blank for EBNA and EgJUB1 systems, respectively. Lane 2 is the positive control for EMSA. EMSA shows direct binding of EgJUB1 to SNBE1 probe in lane 6. EgJUB1 is unable to bind to untransformed yeast and biotinylated mutant SNBE1 (mSNBE1) probe in lane 4 and 5, respectively. Successful binding of 200-fold molar excess of unlabelled SNBE1 (competitor) probe is shown in lane 7. [file 12870_2020_2812_MOESM1_ESM.docx]
