# Supplementary material for: EgJUB1 and EgERF113 transcription factors as potential master regulators of defense response in Elaeis guineensis against the hemibiotrophic Ganoderma boninense
Source: BMC Plant Biol. 2021 Jan 22;21:59. doi: 10.1186/s12870-020-02812-7 (PMC7825162; doi:10.1186/s12870-020-02812-7)

**Additional file 3 : Electrophoretic mobility shift assay (EMSA) of EgERF113 with DRE/CRT probe.** Lane 1 to 3 consist of EBNA control system. Lane 4 to 8 consist of EgERF113 test system. Lane 1 and 8 are the blank for EBNA and EgERF113 systems, respectively. Lane 2 is the positive control for EMSA. EMSA shows direct binding of EgERF113 to DRE/CRT probe in lane 6. EgERF113 is unable to bind to untransformed yeast and biotinylated mutant DRE/CRT (mDRE/CRT) probe in lane 4 and 5, respectively. Successful binding of 200-fold molar excess of unlabelled DRE/CRT (competitor) probe is shown in lane 7.

| **1** | **2** | **3** | **4** | **5** | **6** | **7** | **8** |
| --- | --- | --- | --- | --- | --- | --- | --- |


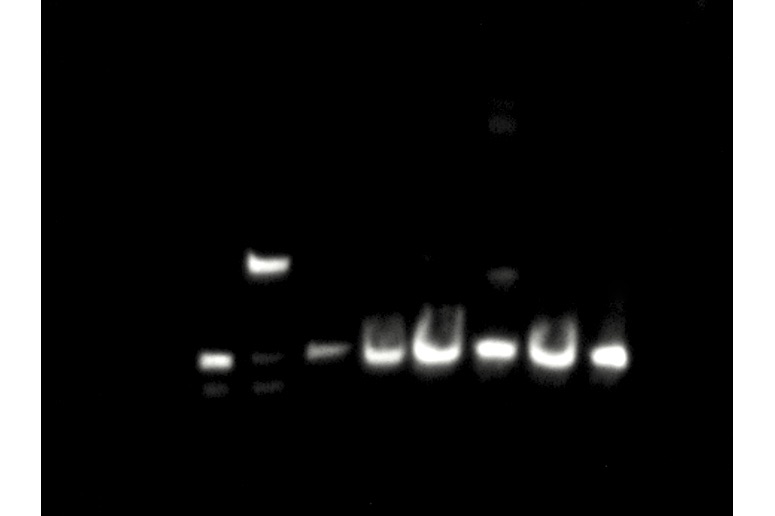

Supplement: Supplementary file 3 — Additional file 3:. Electrophoretic mobility shift assay (EMSA) of EgERF113 with DRE/CRT probe. Lane 1 to 3 consist of EBNA control system. Lane 4 to 8 consist of EgERF113 test system. Lane 1 and 8 are the blank for EBNA and EgERF113 systems, respectively. Lane 2 is the positive control for EMSA. EMSA shows direct binding of EgERF113 to DRE/CRT probe in lane 6. EgERF113 is unable to bind to untransformed yeast and biotinylated mutant DRE/CRT (mDRE/CRT) probe in lane 4 and 5, respectively. Successful binding of 200-fold molar excess of unlabelled DRE/CRT (competitor) probe is shown in lane 7. [file 12870_2020_2812_MOESM3_ESM.docx]
